# Derepression of a single microRNA target causes female infertility in mice

**DOI:** 10.1093/nar/gkaf1357

**Published:** 2026-01-14

**Authors:** Joanna Stefano, Lara E Elcavage, Sue-Jean Hong, David P Bartel, Benjamin Kleaveland

**Affiliations:** Howard Hughes Medical Institute, Cambridge, MA 02142, United States; Whitehead Institute for Biomedical Research, Cambridge, MA 02142, United States; Department of Biology, Massachusetts Institute of Technology, Cambridge, MA 02139, United States; Howard Hughes Medical Institute, Cambridge, MA 02142, United States; Whitehead Institute for Biomedical Research, Cambridge, MA 02142, United States; Department of Biology, Massachusetts Institute of Technology, Cambridge, MA 02139, United States; Harvard-MIT MD-PhD Program, Harvard Medical School, Boston, MA 02115, United States; Howard Hughes Medical Institute, Cambridge, MA 02142, United States; Whitehead Institute for Biomedical Research, Cambridge, MA 02142, United States; Department of Biology, Massachusetts Institute of Technology, Cambridge, MA 02139, United States; Howard Hughes Medical Institute, Cambridge, MA 02142, United States; Whitehead Institute for Biomedical Research, Cambridge, MA 02142, United States; Department of Biology, Massachusetts Institute of Technology, Cambridge, MA 02139, United States; Department of Pathology and Lab Medicine, Weill Cornell Medicine, New York, NY 10065, United States

## Abstract

The miR-200a and miR-200b families control mouse ovulation and are essential for female fertility. The ZEB1 transcription factor is a conserved target of both families and has been implicated as a key player in female fertility at multiple levels. Using gene-edited mice that express a miR-200a/b-resistant form of *Zeb1*, we found that derepression of *Zeb1* in the female pituitary caused decreased production of luteinizing hormone and anovulatory infertility. These phenotypes were accompanied by widespread changes in pituitary gene expression characterized by decreased levels of ZEB1 targets, which include the miR-200a/b microRNAs (miRNAs), as expected from the miR-200a/b–ZEB1 double-negative feedback loop. Also observed were increased levels of mesenchymal genes, neuronal genes, and miR-200a/b targets. These results show that a double-negative feedback loop centered on the miRNA regulation of a single transcription factor can significantly influence the expression of thousands of genes and have dramatic phenotypic consequences.

## Graphical Abstract

**Graphical Abstract:**
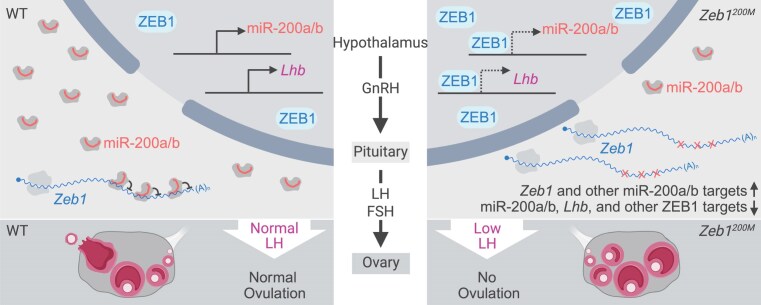

## Introduction

Ovulation disorders are a common cause of female infertility [1, 2]. In mammals, ovulation relies on the coordinated synthesis and secretion of hormones from the hypothalamic–pituitary–ovarian axis. One of these hormones is luteinizing hormone (LH), a ∼25 kDa glycoprotein heterodimer comprised of a unique beta-subunit encoded by the *Lhb* gene and a common alpha-subunit that is shared with follicle-stimulating hormone (FSH), thyroid-stimulating hormone, and chorionic gonadotropin [3]. LH is secreted by gonadotrope cells of the anterior pituitary and stimulates follicle differentiation and steroid hormone biosynthesis in the ovary [4]. During proestrus, an acute burst in LH secretion triggers release of one or more oocytes from the ovary and transforms the empty follicle into a corpus luteum, a structure that supports the fertilized oocyte until placentation occurs [5, 6]. Although mutations in *LHB* are a rare cause of hypogonadism and infertility in both women and men [7–13], abnormally low or high levels of LH are often associated with, and likely contribute to, female infertility arising from dysfunction of the hypothalamic–pituitary–ovarian axis [14]. Given the central role of LH in female fertility and its dysregulation in fertility disorders, the regulatory processes governing LH expression and secretion are of substantial biomedical interest but remain incompletely understood.

A recurring motif in gene regulatory networks is the double-negative feedback loop (DNFL), which consists of two factors that inhibit each other [15]. By amplifying small changes to either factor, mutual inhibition generates a non-linear response to a linear stimulus, which allows some DNFLs to act as ultrasensitive “toggle” switches for bistable systems [16]. One of the best-studied DNFLs centers on the miR-200a/b microRNA (miRNA) families and zinc finger E-box-binding homeobox 1 (ZEB1) and 2 (ZEB2) transcription factors [17, 18]. The evolutionarily related miR-200a and miR-200b families are post-transcriptional repressors that guide Argonaute proteins to the 3′ untranslated regions (UTRs) of target messenger RNAs (mRNAs), including *ZEB1* and *ZEB2*, which accelerates the deadenylation and decay of these mRNAs [19–23]. The ZEB1 and ZEB2 transcription factors bind CAGGTR consensus motifs in the promoters of hundreds of genes and inhibit or activate transcription, depending on the cellular context and interactions with co-repressors and co-activators [24, 25]. ZEB1/2 binding to the promoters of the *Mir200* loci inhibits transcription of the miR-200a/b host genes [20, 26], thereby establishing the miR-200a/b–ZEB1/2 DNFL.

The miR-200a/b–ZEB1/2 DNFL has wide-ranging functions but was first described in human cancer cell lines undergoing epithelial–mesenchymal transition (EMT) [20–23, 26, 27]. In epithelial cells, a ZEB1^low^/miR-200a/b^high^ state maintains a polarized, stationary phenotype. This balance can be shifted by TGF-beta signaling and other factors that stimulate *Zeb1* transcription, leading to direct repression of key epithelial genes, such as *Mir200, Cdh1* (encoding E-cadherin), and *Epcam* (encoding epithelial cell adhesion molecule), and increased expression of mesenchymal markers such as vimentin and alpha-smooth muscle actin [21, 23, 28–31].

Besides its role in EMT, the miR-200a/b–ZEB1/2 DNFL has also been implicated as a key regulatory module in various aspects of reproductive physiology. During puberty, gonadotropin-releasing hormone (GnRH) neurons of the hypothalamus transition from a ZEB1^high^/miR-200a/b^low^ state to a ZEB1^low^/miR-200a/b^high^ state, which is thought to facilitate an increase in GnRH expression required for sexual maturation [32]. An analogous transition has also been observed in the myometrial cells of the uterus during pregnancy; a ZEB1^high^/miR-200a/b^low^ quiescent state throughout gestation is converted to a ZEB1^low^/miR-200a/b^high^ contractile state at the end of pregnancy and during labor [33]. The ZEB1^low^/miR-200a/b^high^ state is also important in the pituitary, as female mice with either deletion of two of the three miR-200b family miRNAs or forced *Zeb1* overexpression in gonadotropes are infertile due to reduced LH expression and a failure to ovulate [34].

Although these experiments demonstrate critical roles for both miR-200a/b and ZEB1 in reproductive physiology, the particular importance of miR-200a/b-mediated repression of *Zeb1/2* or vice-versa is difficult to disentangle with traditional knockout or overexpression approaches because miR-200a/b and ZEB1/2 each have hundreds of targets. Here, we report on the molecular and physiological functions of the miR-200a/b–ZEB1/2 DNFL using *Zeb1* knock-in mice that are resistant to miR-200-mediated repression.

## Materials and methods

### Mouse husbandry

Mice were group-housed in a 12-h light/dark cycle (light between 07:00 and 19:00) in a temperature-controlled room (21.1 ± 1.1°C) at the Whitehead Institute for Biomedical Research with free access to water and food and maintained according to protocols approved by the Massachusetts Institute of Technology Committee on Animal Care. Euthanasia was performed by CO_2_ inhalation.

### Generation of *Zeb1^200^* and *Zeb2^200^* mice

The previously reported *Zeb1^200^* knock-in mice [35] were backcrossed to C57BL/6J mice for at least 15 generations prior to initiating survival and fertility studies. The survival of heterozygous (*Zeb1^200H^*) and homozygous (*Zeb1^200M^*) knock-in mice was confirmed in mouse lines derived from the two independent ES cell clones (6B6 and 3C5) (Supplementary Table S1). The *Zeb1^200H^* knock-in mice derived from clone 6B6 have been deposited at JAX with the MGI ID Zeb1<tm1.1Dpbl>.

Heterozygous *Zeb2^200H^* knock-in mice were generated as follows. To generate the targeting construct, a DNA fragment containing the mouse *Zeb2* locus was subcloned from the bacterial artificial chromosome RP23-454M20. The targeting arms spanned the last exon of *Zeb2*, which includes the entire 3′ UTR (Supplementary Fig. S1C). All eleven miR-200a/b sites were mutated by site-directed mutagenesis. A LoxP-flanked puromycin-resistance gene was used for positive selection, and diphtheria toxin gene (DTA) was used for negative selection. The targeting construct was electroporated into v6.5 mouse embryonic stem cells (genotype 129SvJae × C57Bl/6; male), and 236 colonies were screened by polymerase chain reaction (PCR) and Southern blot (Supplementary Table S8), yielding 21 positive clones. One clone with the first 10 sites mutated (3E3) was injected into C57BL/6J blastocysts and transferred into pseudo-pregnant females. Chimeras were bred with C57BL/6J mice to generate heterozygous progeny, and germline transmission of the mutant allele was verified by PCR. The puromycin-resistance cassette was excised by intercrossing with transgenic CMV-cre mice. Heterozygous knock-in mice were backcrossed to C57BL/6J mice for at least 10 generations. The *Zeb2^200H^* mice derived from clone 3E3 have been deposited at JAX with the MGI IDs Zeb2<tm1.1Dpbl>.

### Genotyping

Genotyping was performed either in-house via end-point PCR or by Transnetyx using real-time PCR. PCR primers can be found in Supplementary Table S8. For in-house genotyping, genomic DNA was extracted from mouse ear snips using the HotSHOT method [36]. PCR was performed using a two-step protocol (98°C for 10 s, 72°C for 10 s) with 1 μl of genomic DNA, Phusion Flash High-Fidelity PCR Master Mix (Thermo Scientific), and 10 pmol of primers, and then analyzed by agarose gel electrophoresis.

### Experimental design and breeding trial

Two cohorts of mice were generated for this study: 39 virgin female mice (*n* = 8 WT, 22 *Zeb1^200H^*, and 9 *Zeb1^200M^*) euthanized at 8–12 weeks for ovary weights, histology, RNA, and protein, and 30 female mice (*n* = 9 WT, 10 *Zeb1^200H^*, and 11 *Zeb1^200M^*) that took part in a breeding trial and were subsequently euthanized at 27–40 weeks for histology, RNA, and serum hormones. The virgin female cohort was generated using both clonal lines (17 animals from 3C5 and 22 animals from 6B6), and littermates were cohoused after weaning until euthanasia. The breeding trial cohort was also generated using both clonal lines (17 animals from 3C5, 11 animals from 6B6/3C5 intercross, and 2 animals from 6B6) and singly housed for estrus cycle staging prior to and after the breeding trial.

In the virgin cohort, female mice (8–12 weeks) were subjected to estrous cycle staging by vaginal cytology. At the first instance of proestrus, mice were euthanized, and reproductive tissues were collected for histology and/or RNA and protein extraction, as described below. In the breeding trial cohort, female mice (8–11 weeks) were subjected to 3 weeks of estrous cycle staging. Of the 30 mice initially selected for this cohort, 29 completed at least one estrus cycle and were included in the subsequent breeding trial. One WT mouse never cycled and was excluded from further studies. Breeding was initiated by adding a WT C57Bl/6J male mouse to each cage. During the 12-week breeding trial, animals were assessed daily for visible pregnancies and new litters. For half of the mice in the trial, including all *Zeb1^200M^* females, breeding was extended an additional 5 weeks, although no additional litters were produced by *Zeb1^200M^* females. Only the results from the first 12 weeks of breeding are plotted in Fig. 2A. At the conclusion of the trial, male mice were removed from cages and, after 20 or more days, females were again staged for estrous cycle. At the first instance of proestrus, mice (27–40 weeks of age) were euthanized, blood was collected for serum hormone measurements, and reproductive tissues were collected for histology and/or RNA extraction, as described below.

### Vaginal cytology

To determine the stages of the estrous cycle, vaginal cytology specimens were collected for 21–23 consecutive days, beginning at 8–11 weeks of age, using a cotton-tipped swab (Puritan Medical Product Company) wetted with normal saline solution (0.9% NaCl) and inserted into the vagina of the restrained mouse. The swab was gently turned and rolled against the vaginal wall and then removed. Cells were transferred to a dry glass slide by rolling the swab across the slide. The slide was air-dried and then stained with Three-Step Stain Kit (Richard-Allan Scientific) for 45 seconds. The slides were rinsed with distilled water, air-dried, and viewed under bright-field illumination at 20× magnification. The stages of the estrous cycle were determined based on the overall cellularity and the presence of nucleated epithelial cells (proestrus), cornified and anucleated epithelial cells (estrus), and/or leukocytes (diestrus), as described previously [37]. One estrous cycle was defined as the sequential appearance of all stages, regardless of time spent in each. As it is not possible to know when the first stage began, estrous cycle lengths were calculated for each animal beginning with the appearance of the second stage, and the percentage of time spent in each stage was determined for each completed cycle. For all genotypes, animals completed an average of ∼2 cycles during the assessment period.

### Hormone measurements

To determine circulating hormone levels, ∼750 μl of blood was collected by cardiac puncture of the ventricle while animals were under terminal anesthesia, and serum LH and FSH concentrations were measured using immunofluorometric assays as described previously [38, 39]. Samples were collected from 27–40-week-old female mice (*n* = 8 WT, 10 *Zeb1^200H^*, and 11 *Zeb1^200M^*) during proestrus, as determined by vaginal cytology.

### Histology

All tissues were collected from mice in proestrus, as determined by vaginal cytology. One ovary per animal was fixed in 10% buffered formalin (Sigma) for 24 h and stored in 70% ethanol. Paraffin-embedded ovaries were serially sectioned and stained with hematoxylin and eosin. For each section, two individuals independently counted the number of early/primary follicles, defined as having fewer than two complete layers of granulosa cells, mature/secondary follicles, defined as having two or more complete layers of granulosa, and corpus luteum. These counts were summed for 2–8 sections taken from different levels of each ovary and then divided by the total area of those sections (mm^2^).

### RNA extraction

Total RNA was extracted from the pituitary, hypothalamus, and ovary tissues collected from WT, *Zeb1^200H^*, and *Zeb1^200M^* mice using TRI Reagent (Thermo Fisher) according to the manufacturer’s protocol with the following modifications. Mouse tissues were rapidly dissected after euthanasia and flash frozen in Eppendorf tubes in liquid N_2_. Tissue was transferred to a 15 or 50 ml conical tube, 1–2 ml of TRI Reagent was added, and the tissue was homogenized with a TissueRuptor (Qiagen) and disposable probes. Samples were transferred to Eppendorf tubes (1 ml per tube) and phase-separated with 200 μl chloroform (J.T. Baker Analytical). After isopropanol precipitation and two 70% ethanol washes, total RNA was resuspended in water.

### RNA-seq and analysis

For all samples in the virgin female cohort (*n* = 3–4 biological replicates per genotype), the Watchmaker RNA Library Prep Kit (Watchmaker Genomics) was used to generate stranded RNA-seq libraries. Prior to library preparation, ribosomal RNA depletion was performed using QIAseq FastSelect –rRNA HMR kit (Qiagen) and 0.5 μg of hypothalamus, pituitary, or ovary total RNA, according to manufacturer’s instructions. Samples were then carried forward to the First Strand Synthesis step of the Watchmaker RNA Library Prep Kit. Libraries were sequenced on the Illumina NovaSeq platform with 50-nt paired-end reads.

For all samples in the breeding trial cohort (*n* = 3–4 biological replicates per genotype), the TruSeq RNA Sample Prep Kit v2 (Illumina) was used to generate unstranded, poly(A)-selected RNA-seq libraries. Briefly, 1 μg of hypothalamus, pituitary, or ovary total RNA was poly(A) enriched and then reverse transcribed into double-strand complementary DNA (cDNA). The cDNA samples were fragmented, end-repaired, and polyadenylated before ligation of TruSeq adapters containing an index sequence for multiplex sequencing. Multiplexing fragments containing TruSeq adapters on both ends were selectively enriched with 15 cycles of PCR. All libraries were sequenced on the Illumina HiSeq 2000 platform with 50-nt single-end reads.

Differential gene expression analysis of RNA-seq data was performed as follows. Reads were aligned to the mouse genome (mm10) using STAR v2.7.1a [40] with the parameters “–outFilterType BySJout –outSAMtype BAM SortedByCoordinate –readFilesCommand zcat –outFilterIntronMotifs RemoveNoncanonicalUnannotated.” For pituitary and hypothalamus libraries from the breeding trial cohort, aligned reads were assigned to genes using htseq-count v0.9.1 [41] with the parameters “-m union -s no” and annotations from Ensembl (Mus_musculus.GRCm38.87.gtf; downloaded May 15, 2017), count files were merged to generate tables for each tissue organized by genotype, and differential expression was determined using DESeq2 v1.18 (pituitary and hypothalamus from the breeding trial cohort) with the parameter “betaPrior = FALSE” and without the lfcShrink function. For ovary libraries from the breeding trial cohort and all libraries from the virgin female cohort, aligned reads were assigned to genes using featureCounts [42] with the parameter “-s 0” (ovary libraries from breeding trial cohort) or “-s 2” (all libraries from virgin female cohort), and annotations from Gencode (m38.p6 gencode.vM25.basic.annotation.gtf; downloaded 7/10/24). Count files were merged as described earlier, and differential expression was determined using DESeq2 v1.38.3 without the lfcShrink function [43]. The DESeq2 outputs for each comparison are provided in Supplementary Table S2 and S3.

RNA-seq browser tracks were visualized in the UCSC Genome Browser and IGV v2.16.1 [44, 45]. For counting *pri-miR-200* species, reads mapping to *ENSMUSG00000086549* (miR-200b∼429 cluster) and *ENSMUSG00000087327* (miR-200c–141 cluster) were used.

For analysis of intron expression in the pituitary, STAR-aligned reads were assigned to gene introns using featureCounts [42] with the parameter “-s 0” (breeding trial cohort) or “-s 2” (virgin female cohort), and annotations which were generated by loading the m38.p6 gencode.vM25.basic.annotation.gtf into the UCSC Genome Browser and downloading the corresponding intron structure information. For each gene, all introns were combined into a single annotation. Differential expression analysis of intron-mapping reads was performed using DESeq2 v1.38.3 without the lfcShrink function [43]. The DESeq2 outputs for each comparison are provided in Supplementary Tables S4 and S5.

For RNA-seq plots, results for only RNAs with a mean normalized CPM > 1 in the control samples (either WT or *Zeb1^200H^*, depending on the comparison) and log_2_ fold changes ≤ 5 and ≥ –5 are shown. A small fraction of differentially expressed genes (DEGs) in each pair-wise comparison (<4% of pituitary DEGs, <2% of hypothalamus DEGs, and <10% of ovary DEGs) were not plotted because of low counts and/or log_2_ fold changes that exceeded the bounds listed earlier. Linear regression was performed on genes with an average of at least 50 raw read counts across samples.

Gene set enrichment analysis (GSEA) was performed using WebGestalt [46] and Gene Ontology functional databases for biological processes and cell components with the following parameters: minimum number of IDs in category, 10; maximum number of IDs in category, 1000; significance level, FDR < 0.05; permutations, 1000. The input for GSEA was a list of *M. musculus* gene IDs with WT pituitary expression >1 CPM ranked by STAT, which is the log_2_ fold change divided by log_2_ fold change standard error. Redundancy of gene sets was reduced with the weighted set cover option.

Principal component analysis was performed on raw RNA sequencing (RNA-seq) read counts for all *Zeb1^200M^, Zeb1^200H^*, and WT samples of each tissue type. The Seurat functions NormalizeData, FindVariableFeatures (selecting the default of 2000 most variable genes), and ScaleData were implemented prior to principal component analysis (http://www.satijalab.org/seurat). The Seurat function RunPCA was used with the number of principal components equal to the total number of samples minus 1.

### Small-RNA sequencing and analysis

For all samples in the virgin female cohort, small-RNA-seq libraries were prepared with 2 μg of total RNA from the pituitary (*n* = 3–4 samples per genotype). In addition, 0.5 fmoles of miR-427–5p (*Xenopus tropicalis*) and 0.5 fmoles of lsy-6–3p (*Caenorhabditis elegans*) were added as spike-ins. From each sample, RNAs that co-migrated within the range of 18 and 32 nt radiolabeled internal standards were isolated on a 15% polyacrylamide urea gel. Purified RNA was ligated to a preadenylated 3′ adapter (AppNNNNTCGTATGCCGTCTTCTGCTTGddC) using T4 RNA ligase 2 KQ mutant (NEB) in a reaction supplemented with 10% polyethylene glycol (PEG 8000, NEB). To reduce ligation biases, this 3′ adapter had four random-sequence positions at its 5′ end. After gel purification on an 8% polyacrylamide urea gel, RNA was ligated to a 5′ adapter (GUUCAGAGUUCUACAGUCCGACGAUCNNNN) using T4 RNA ligase I (NEB) in a reaction supplemented with 10% PEG. To reduce ligation biases, this adapter had four random-sequence positions at its 3′ end. After gel purification on an 8% polyacrylamide urea gel, RNA was reverse transcribed with SuperScript III (Invitrogen), and the cDNA was amplified ∼12 cycles with KAPA HiFi DNA polymerase (Kapa Biosystems). Amplified cDNA was purified using a 1.8X AmpureXP (Beckman Coulter) bead cleanup, followed by agarose gel on the PippinHT. Libraries were sequenced on the Illumina NovaSeq platform with 50 nt paired-end reads. For all samples in the breeding trial cohort, small-RNA-seq libraries were each prepared with 0.5 μg of total RNA from the pituitary (*n* = 3–4 samples per genotype). To minimize loss due to low starting input, 3 μg of total RNA from the budding yeast *Naumovozyma castelli* was added to each sample prior to size selection. In addition, 0.5 fmoles of miR-427–5p (*X. tropicalis*) and 0.5 fmoles of miR-14 (*Drosophila melanogaster*) were added as spike-ins. Size selection, adapter ligation, reverse transcription, and cDNA amplification was performed as described earlier. Amplified DNA was purified on a 3% metaphor agarose gel and submitted for sequencing. Libraries were sequenced on the Illumina HiSeq platform with 50 nt single-end reads. A step-by-step protocol for constructing libraries for small-RNA-seq is available at http://bartellab.wi.mit.edu/protocols.html.

Reads were trimmed of adaptor sequence using cutadapt [47] and filtered using fastq_quality_filter (FastX Toolkit; http://hannonlab.cshl.edu/fastx_toolkit/) with the parameters “-q 30 –p 100” to ensure that all bases had an accuracy of 99.9%. For the libraries prepared from the virgin female cohort, the first 19 nt of each read were matched to a dictionary of miRNA sequences downloaded from TargetScanMouse Release 7, requiring no mismatches between the read and the miRNA dictionary. For the libraries prepared from the breeding trial cohort, the first 18 nt of each read were matched to a dictionary of miRNA sequences based on annotations in miRbase_v20. Reads mapping to the internal size standards and spike-in miRNAs were removed for further analysis. Differential expression analysis was performed using DESeq2 v1.38.3 without use of the lfcShrink() function (virgin female cohort) or DESeq2 v1.18 with the parameter “betaPrior = FALSE” and without the lfcShrink function (breeding trial cohort) [43]. Counts per million (CPM) were calculated by summing across DESeq2 normalized miRNA counts within each sample, then multiplying by 10^6^. The DESeq2 outputs for each comparison are provided in Supplementary Tables S6 and S7.

For small-RNA-seq plots, only miRNAs with a mean normalized CPM > 1 in WT for virgin female samples and CPM > 10 in WT for breeding trial samples are shown. Linear regression was performed on genes with an average of at least 50 raw read counts across samples.

### miRNA target analysis

miRNA target predictions were downloaded from TargetScanMouse Release 8.0 [48]. Differential expression data (DESeq2 output) was analyzed for repression of genes predicted to be miRNA targets. Only genes with an average of at least 50 raw read counts across samples were considered. Three sets of predicted targets were analyzed: all predicted targets, conserved predicted targets, and top predicted targets. Top predicted targets were defined as the top 10% of all predicted targets based on cumulative weighted context++ scores [49]. Each of the three sets of targets was compared to a control group of transcripts not predicted to be targets of the miRNA family in question. This nontarget set was selected as follows. First, 3′ UTR sequences from TargetScanMouse Release 8.0 (https://www.targetscan.org/cgi-bin/targetscan/data_download.cgi?db=mmu_72; downloaded on 7/10/24) were used to group genes into 10 bins based on 3′ UTR length. Next, for each target, one transcript not predicted to be a target of the miRNA family was selected, with replacement, from the corresponding UTR bin. For each target set, the distribution of log_2_ fold changes (computed using DESeq2) in *Zeb1^200M^* or *Zeb1^200H^* samples relative to WT samples was compared to the distribution of log_2_ fold changes of the 3′ UTR length-matched nontarget cohort, and statistical significance was determined using a Mann–Whitney test. The degree of repression is represented by subtracting the median log_2_ fold change of the target set from the median log_2_ fold change of its 3′ UTR length-matched nontarget set. The analysis described earlier was repeated 20 additional times for each target set, and the mean difference in median log_2_ fold changes and the median *P* value across the 21 replicates were reported. For cumulative distribution functions, only the nontarget set corresponding to the “all targets” set is shown for simplicity.

### Quantification and statistical analysis

Graphs were generated in GraphPad Prism 7–10, R, or Python, and statistical analyses were performed using Excel, GraphPad, R, or Python. Statistical parameters, including the value of *n*, statistical test, and statistical significance (*P* value), are reported in the figures and their legends. For studies involving mouse tissues, replicates refer to samples derived from different mice. No statistical methods were used to predetermine sample size. Statistical tests were selected based on the desired comparison.

One-way analysis of variance (ANOVA) was used to assess significance when comparing fertility measurements between three genotypes; significant ANOVA results were followed by a post-hoc Kruskal–Wallis test. Two-way ANOVA was used to assess significance when comparing ZEB1 protein expression by western blot using genotype and biological replicate number as the two factors; significant ANOVA results were followed by a Tukey’s multiple-comparison test. Linear mixed-effects model ANOVA was used to assess significance when comparing ovary weights between three genotypes using genotype as a fixed effect, animal ID as a random effect, and ovary weight as a dependent variable; significant ANOVA results were followed by a Tukey’s multiple comparison test. For differential expression of global measurements (RNA-seq, small-RNA-seq), the DESeq2 software generated adjusted *P* values using the Wald test with the Benjamini–Hochberg procedure to correct for multiple-hypothesis testing. The Mann–Whitney test was used to compare cumulative distributions of mRNA fold changes between two gene sets. The Fisher’s exact test was used to determine the significance of overlap between gene sets.

### Western blotting

Mouse pituitary glands (*n* = 3 per genotype) were dissected and flash frozen in liquid nitrogen. Frozen tissues were lysed in 50 μl 1× radioimmunoprecipitation assay (RIPA) buffer, supplemented with 1× Halt Protease & Phosphatase Inhibitor Cocktail (Thermo Scientific 1861280) and 0.001 M DTT, in a water bath sonicator until homogenized. Total protein was quantified using the Pierce Detergent Compatible Bradford Assay (Thermo Scientific 23246).

Samples were heated for 10 min at 65°C in 1× NuPAGE LDS Sample Buffer (Invitrogen NP0008) supplemented with 2.5% beta-mercaptoethanol. Twenty micrograms total protein per sample was resolved on a NuPAGE Bis-Tris, 4%–12% protein gel in 1× 2-(N-morpholino)ethanesulfonic acid (MES) sodium dodecyl sulfate running buffer (Thermo Scientific NP0002). Protein was transferred to a PVDF membrane (Thermo Scientific 88520) in 1× NuPAGE Transfer Buffer (Thermo Scientific NP00061) with 10% methanol. The membrane was blocked in 1× Tris-buffered saline with Tween-20 (TBST) with 5% bovine serum albumin (BSA) and incubated with primary antibody in 2.5% BSA overnight at 4°C. Primary antibodies used were rabbit anti-ZEB1 (CST 3396; 1:1000) and mouse anti-GAPDH (Proteintech 60004-1; 1:50 000). The membrane was washed and incubated with the secondary antibodies IRDye 680RD Goat anti-Rabbit (LICORbio 926-68 071) and IRDye 800RD Goat anti-Mouse (LICORbio 926–32 210). The membrane was imaged on a LICOR Odyssey CLx, and bands were quantified using average local background subtraction in Image Studio.

## Results

### miR-200a/b represses *Zeb1* in the female pituitary

**Figure 1.**
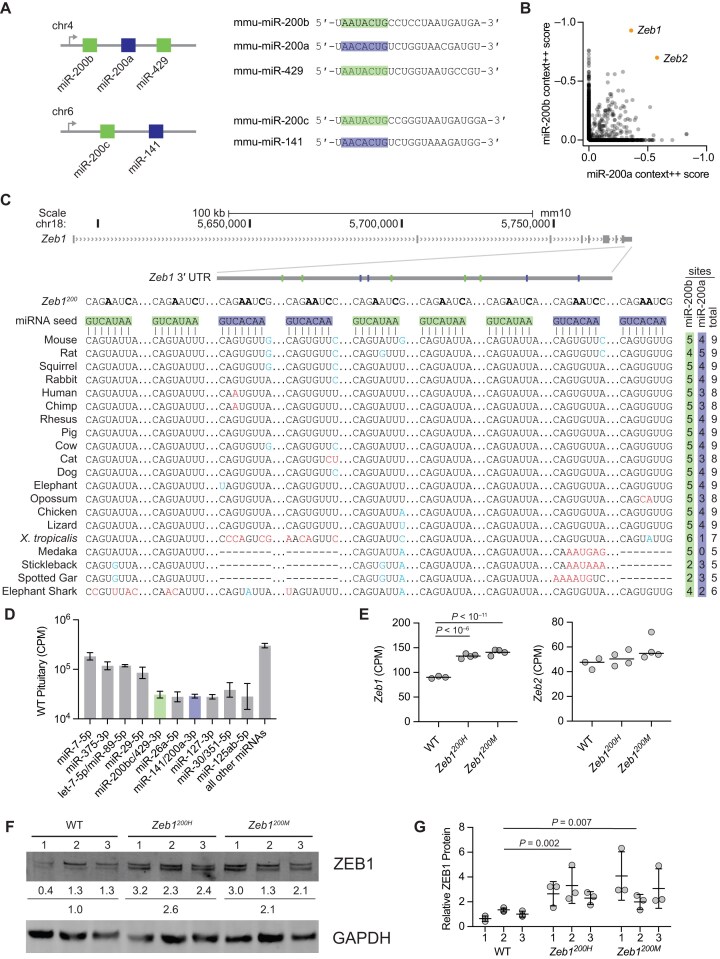
*Zeb1* is regulated by the miR-200 superfamily in the mouse pituitary. (**A**) The miR-200 superfamily members are expressed from two miRNA gene clusters. Diagrammed on the left are the miR-200b∼200a∼429 and miR-200c∼141 loci on murine chromosomes 4 and 6, respectively. Shown on the right are the mature sequences of each member of the miR-200 superfamily. The miR-200a/141 family seed sequence (blue) differs from the miR-200b/c/429 family seed sequence (green) by a single nucleotide at position 4 (counting from the 5′ end). (**B**) Predicted targets of miR-200b/c/429 and miR-200a/141 in mice. Plotted are TargetScan7 cumulative weighted context++ scores for both miRNA families, with each circle representing a gene that is predicted as a target of miR-200b and/or miR-200a. Targets predicted with higher confidence have lower context++ scores. As expected for two miRNA families with distinct seed sequences, most predicted targets of miR-200b are not predicted targets of miR-200a and vice versa. (**C**) Organization of the murine *Zeb1* locus. The *Zeb1* gene model (gray boxes, exons; > > >, introns) is depicted with an inset of the 3′ UTR indicating the location of the binding sites for miR-200b (green boxes) and miR-200a (blue boxes). Diagramed below that is the pairing of five miR-200b sites and four miR-200a sites and Multiz alignments for each site in 20 vertebrate species (red denotes a nucleotide change relative to the mouse sequence that disrupts pairing, blue denotes a nucleotide change that maintains at least a 7-mer seed site, some of which convert a miR-200b site to a miR-200a site or vice versa). Also shown are the mutant sites of the *Zeb1*^200^ locus, indicating the substitutions (bold) that were introduced in each binding site to disrupt miRNA binding. The number of conserved miR-200b, miR-200a, and total sites are listed to the right of the alignment. (**D**) miRNA expression in WT pituitary. Plotted are the mean CPM mapped miRNAs of the 10 highest expressed miRNA families and all remaining miRNAs, as determined by small-RNA-seq, in WT pituitary from 8–12-week-old female mice (error bars, s.d.; *n* = 3–4). (**E**) Influence of *Zeb1^200^* alleles on *Zeb1* and *Zeb2* expression in the pituitary. Plotted are the CPM mapped reads for *Zeb1* and *Zeb2*, as determined by RNA-seq, in WT, *Zeb1^200H^*, and *Zeb1^200M^* pituitary from 8–12-week-old female mice (black line, mean; *n* = 3–4 per genotype). Benjamini–Hochberg adjusted *P* values, as determined by a Wald test (DESeq2), are shown for *P* values < .05. (**F**, **G**) Influence of *Zeb1^200^* alleles on ZEB1 protein expression in the pituitary. A representative western blot measuring ZEB1 levels in the pituitary of 8–12-week-old female mice (*n* = 3 per genotype) is shown in panel (F). In each lane, the intensity of ZEB1 was normalized to that of GAPDH and reported relative to the average of the three WT samples. The results for three technical replicates (separate gels) are shown in panel (G). Biological replicates, indicated as numbers along the *x*-axis, correspond to the numbered lanes in panel (F) (black line, mean; error bars, s.d.). Only statistically significant changes (adjusted *P* value < .05) for all pairwise comparisons are indicated (two-way ANOVA using the factors genotype and biological replicate, with Tukey’s multiple comparisons test).

*Zeb1* and *Zeb2* are among the top predicted targets of the miR-200a/b families in both mice and humans (Fig. 1A and B and Supplementary Fig. S1A), owing to an unusually large number of conserved sites for each miRNA family residing in the 3′ UTRs of each gene [49]. In addition, both *Zeb1* and *Zeb2* have been experimentally validated as miR-200a/b targets in diverse cancerous and noncancerous cells [20–23, 26, 27, 35, 50]. To investigate the physiologic role of miR-200a/b–mediated repression of *Zeb1* and *Zeb2* in mammals, we took advantage of the previously published *Zeb1* knock-in mouse (*Zeb1^200^*), which has point mutations in each of the nine predicted miR-200a/b binding sites in the *Zeb1* 3′ UTR (Fig. 1C), and also generated a *Zeb2* knock-in mouse (*Zeb2^200^*), replacing the wild-type (WT) *Zeb2* 3′ UTR with a mutant 3′ UTR in which 10 of the 11 predicted miR-200a/b binding sites are each disrupted by 2–3 point mutations—thus creating a miR-200a/b–resistant *Zeb2* allele (Supplementary Fig. S1B and C). This knock-in approach offers advantages over more broadly used knockout and overexpression methods for interrogating the relationship between a miRNA and its target. Namely, this approach: (i) directly affects only one out of potentially hundreds of miRNA targets, (ii) evaluates regulation of a target by all miRNA family members without requiring a priori knowledge about which miRNA family members are expressed in a particular cell/tissue, and (iii) produces physiologically relevant increases in the target RNA and protein. Both the miR-200a/b–resistant *Zeb1* mice and the miR-200a/b–resistant *Zeb2* mice were viable and grossly normal (Supplementary Table S1); in this study, we focus on the striking phenotypic and molecular findings in the miR-200a/b–resistant *Zeb1* mice.

To determine whether the mutant *Zeb1* allele was no longer susceptible to miR-200a/b-mediated repression, we examined *Zeb*1 expression in the female pituitary, a tissue with abundant miR-200a/b expression (Fig. 1D) [34]. RNA-seq of pituitaries collected from virgin female mice staged for proestrus at 8–12 weeks of age confirmed that disruption of miR-200a/b-mediated repression of *Zeb1* led to modest but significant increases in *Zeb1* mRNA—1.3-fold in heterozygous knock-in mice (*Zeb1^200H^*) and 1.4-fold in homozygous knock-in mice (*Zeb1^200M^*)—without affecting RNA splicing or 3′ end formation (Fig. 1E and Supplementary Fig. S1D). Similar *Zeb1* derepression—1.3-fold in *Zeb1^200H^* and 1.5-fold in *Zeb1^200M^*—was detected in pituitaries from older females at 28–36 weeks of age, indicating that miR-200a/b-mediated repression persists throughout adulthood (Supplementary Fig. S1E). The low magnitude of derepression was consistent with the ≤2-fold effects previously observed in reporter assays and in pancreatic islets from *Zeb1^200M^* mice [21–23, 35, 51].

To assess ZEB1 protein expression, we first optimized semi-quantitative western blotting across a four-fold range of total protein inputs (10–40 μg) from mouse pituitary (Supplementary Fig S1F). We then quantified ZEB1 levels in 20 μg total protein collected from pituitaries of 8–12-week-old virgin mice staged for proestrus. ZEB1 protein expression was increased 2.8-fold in heterozygous knock-in mice (*Zeb1^200H^*) and 3.0-fold in homozygous knock-in mice (*Zeb1^200M^*) compared to WT mice (Fig. 1F and G). The fold changes in ZEB1 protein were higher than the fold changes in *Zeb1* mRNA, raising the possibility of miR-200-mediated translational repression, although given the variability in these measurements, we cannot exclude measurement error as a source of these differences.

### Loss of miR-200 regulation of *Zeb1* causes impaired female fertility

During the course of maintaining the colony, we noted no fertility defects in adult *Zeb1^200M^* males. However, the adult *Zeb1^200M^* female mice were subfertile. Here, we focused on this sex-specific fertility phenotype.

**Figure 2.**
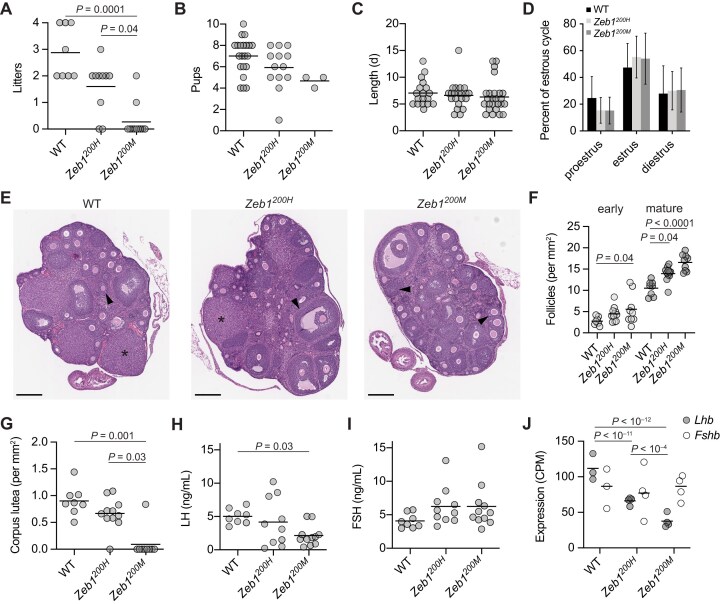
miR-200a/b-resistant *Zeb1* mice have decreased fertility. (**A, B**) Influence of *Zeb1^200^* alleles on number and size of litters. Plotted in panel (A) are the number of litters per female for WT, *Zeb1^200H^*, and *Zeb1^200M^* female mice mated with WT male mice for 12 weeks (black line, mean; *n* = 8–11 mice per genotype). Plotted in panel (B) are the number of pups per litter for the same cohort. Only statistically significant changes (adjusted *P* value < .05) for all pairwise comparisons are indicated (ANOVA with Kruskal–Wallis test). Statistical testing for differences in litter size between *Zeb1^200M^* mice and the other two genotypes lacked sufficient power due to the small number of litters produced by *Zeb1^200M^* females. (**C, D**) Influence of *Zeb1^200^* alleles on estrus cycle length and stage. Plotted in panel (C) are the lengths in days for each estrus cycle, as determined by vaginal cytology, for WT, *Zeb1^200H^*, and *Zeb1^200M^* female mice (black line, mean; *n* = 18–23 estrus cycles from 8–11 mice per genotype). Plotted in panel (D) are the mean percentages of time (in days) spent in proestrus, estrus, and diestrus for the same cohort (error bars, s.d.). No statistically significant changes (adjusted *P *< .05) were detected (ANOVA with Kruskal–Wallis test). (**E**–**G**) Influence of *Zeb1^200^* alleles on ovarian folliculogenesis. Shown in panel (E) are hematoxylin and eosin stained sections of ovary from 8–12-week-old WT, *Zeb1^200H^*, and *Zeb1^200M^* female mice (arrowhead, secondary/mature follicle; asterisk, corpus luteum; scale bar, 300 μm). The number of early/primary and mature/secondary follicles per mm^2^ ovary section and the number of corpus luteum per mm^2^ ovary section are plotted in panels (F) and (G), respectively, for the indicated genotypes (black line, mean; *n* = 8–11 mice per genotype). Only statistically significant changes (adjusted *P* value < .05) for all pairwise comparisons are indicated (ANOVA with Kruskal–Wallis test). (**H**–**I**) Influence of *Zeb1^200^* alleles on circulating LH and FSH levels. Plotted are concentrations of LH (H) and FSH (I) in serum harvested from 27–40-week-old WT, *Zeb1^200H^*, and *Zeb1^200M^* female mice at the conclusion of the breeding trial (black line, mean; *n* = 8–11 mice per genotype) during proestrus, as determined by vaginal cytology. Only statistically significant changes (adjusted *P* value < .05) for all pairwise comparisons are indicated (ANOVA with Kruskal–Wallis test). (**J**) Influence of *Zeb1^200^* alleles on *Lhb* and *Fshb* expression in the pituitary. Plotted are the CPM-mapped reads for *Lhb* and *Fshb*, as determined by RNA-seq, in the pituitary of 8–12-week-old WT, *Zeb1^200H^*, and *Zeb1^200M^* female mice staged for proestrus (black line, mean; *n* = 3–4 per genotype). Benjamini–Hochberg adjusted *P* values, as determined by a Wald test (DESeq2), are shown for *P* values < .05.

When paired with C57Bl/6J males for 12 weeks, 9 of 11 *Zeb1^200M^* females failed to produce any litters (Fig. 2A). The *Zeb1^200M^* females that did become pregnant had fewer litters than WT or *Zeb1^200H^* females (Fig. 2A), and *Zeb1^200M^* litters tended to be smaller (Fig. 2B). *Zeb1^200M^* females had normal body weights and robust estrous cyclicity (Fig. 2C and D; Supplementary Fig. S2A and B), indicating that the subfertility was likely not due to abnormal development of the hypothalamic–pituitary–ovarian axis.

To identify the basis of the impaired fertility in *Zeb1^200M^* females, we examined the ovaries of 8–12-week-old virgin mice staged for proestrus. *Zeb1^200M^* ovaries were 24.6% smaller than WT ovaries (Supplementary Fig. S2C), and histology revealed follicles at all stages of development with greater numbers of mature follicles compared to WT ovaries (Fig. 2E and F). However, the number of corpus lutea was greatly diminished (Fig. 2G). Because corpus lutea formation depends on ovulation, the paucity of corpus lutea and increased number of follicles in *Zeb1^200M^* ovaries suggested ineffective ovulation. Although ovaries collected from older mice at the conclusion of the breeding trial tended to contain fewer follicles than those from the younger mice, *Zeb1^200M^* ovaries in this older cohort again displayed a paucity of corpus lutea (Supplementary Fig. S2E and S2F).

The reduction in corpus lutea in *Zeb1^200M^* ovaries raised the possibility that LH synthesis and/or secretion might be dysregulated. We measured serum levels of LH and FSH during proestrus, when LH levels typically surge. Indeed, circulating LH levels were decreased 2.4-fold in *Zeb1^200M^* females, whereas circulating FSH levels were not significantly changed (Fig. 2H,I). *Zeb1^200H^* females, which are fertile, had normal levels of LH and FSH. To determine whether the decreased circulating LH might be caused by decreased LH production in the pituitary, we examined *Lhb* expression in our pituitary RNA-seq data. *Lhb* levels were reduced 1.8-fold in *Zeb1^200H^* pituitaries and 3.2–4.5-fold in *Zeb1^200M^* pituitaries (Fig. 2J and Supplementary Fig. S2G), consistent with previous reports showing that the *Lhb* promoter, which contains at least four E-box motifs, is bound and repressed by ZEB1 [34, 52].

In theory, the decreased expression of *Lhb* in *Zeb1* mutant pituitaries could be explained by either a reduction in the number of gonadotrope cells or a reduction in *Lhb* expression within the gonadotrope lineage. *Fshb*, which encodes the unique beta-subunit of FSH, is also specifically expressed in gonadotropes. Unlike *Lhb, Fshb* levels were not affected in *Zeb1* mutants (Fig. 2J and Supplementary Fig. S2G), indicating that the reduction in *Lhb* was due to reduced *Lhb* expression within the gonadotrope lineage and not a loss of gonadotropes.

### Massive gene expression changes accompany derepression of *Zeb1*

To gain a better understanding of the impact of elevated ZEB1 on pituitary gene expression, we performed differential expression analysis of RNA-seq data generated from two independent cohorts of female mice staged for proestrus. The first cohort was composed of virgin 8–12-week-old mice (Supplementary Tables S2 and S4), and the second was composed of 28–36-week-old mice that completed the breeding trial (Supplementary Tables S3 and S5).

**Figure 3.**
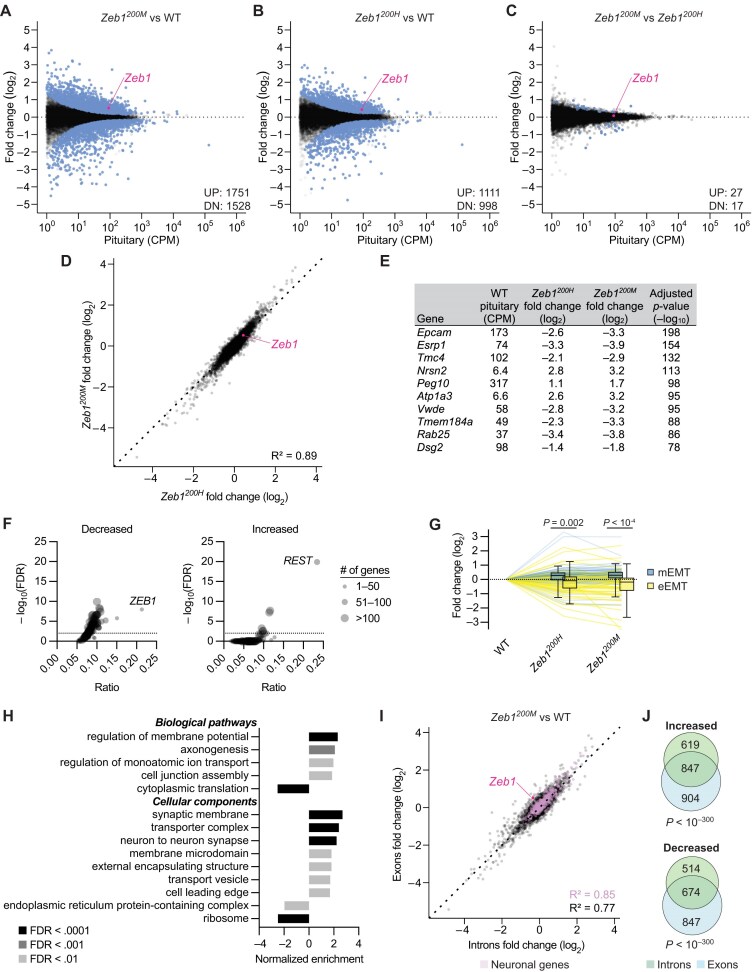
Widespread gene expression changes induced by modest derepression of *Zeb1* in virgin female mice. These analyses are of RNA-seq results from pituitary of 8–12-week-old WT, *Zeb1^200H^*, and *Zeb1^200M^* female mice staged for proestrus (*n* = 3–4 per genotype). (**A**–**C**) The influence of *Zeb1^200^* alleles on RNA levels in the pituitary. Shown are fold changes in mean RNA levels for *Zeb1^200M^* relative to WT (**A**), *Zeb1^200H^* relative to WT (**B**), and *Zeb1^200M^* relative to *Zeb1^200H^* (**C**), plotted as a function of expression in WT pituitary. Each circle represents a unique mRNA or noncoding RNA, showing results for all RNAs expressed above 1 CPM mapped reads. Blue circles indicate DEGs as determined by DESeq2 (adjusted *P* value < .05), and the number of significantly increased (UP) and decreased (DN) genes is indicated. (**D**) Correlation of RNA fold changes between *Zeb1^200^* alleles. Plotted are fold changes of *Zeb1^200M^* and *Zeb1^200H^* pituitary relative to WT pituitary. (**E**) Top 10 DEGs, ranked by adjusted *P* value. For each gene, mean WT pituitary expression and fold changes in *Zeb1^200H^* and *Zeb1^200M^* pituitary relative to WT pituitary are shown. (**F**) Overrepresentation of transcription factor binding sites in DEGs. Plotted are the ratio of genes with transcription factor binding sites that are also increased or decreased between *Zeb1^200M^* and WT pituitary (adjusted *P* value < .05) versus the false discovery rate, as determined by ChEA3. Each circle represents the high-confidence targets of an individual transcription factor assembled from ENCODE ChIP-Seq data. (**G**) The influence of *Zeb1^200^* alleles on genes associated with EMT. Plotted are the fold changes of 46 epithelial EMT (eEMT) genes and 62 mesenchymal EMT (mEMT) genes in *Zeb1^200M^* and *Zeb1^200H^* pituitary relative to WT pituitary. Statistical significance of the difference between eEMT genes and mEMT genes for each genotype are indicated (unpaired *t*-test). (**H**) GSEA of *Zeb1^200M^* pituitary. Plotted are normalized enrichment scores for all statistically significant (FDR < 0.01) biological pathway and cell component gene ontologies, as determined by WebGestalt [46]. (**I**) Correlation of RNA exon and intron fold changes in *Zeb1^200M^* pituitary. Plotted are exonic fold changes of *Zeb1^200M^* relative to WT pituitary versus intronic fold changes for the same comparison. The correlation coefficient (Pearson *R*^2^) is indicated, and neuronal genes are highlighted in lavender. (**J**) Overlap of DEGs (adjusted *P* value < .05) based on exonic read counts or intronic read counts. Shown are Venn diagrams for genes that are either increased or decreased in *Zeb1^200M^* relative to WT pituitary based on mapping to exons or introns. The significance of the overlap is indicated (Fisher’s exact test).

In *Zeb1^200M^* pituitary from virgin females, 3279 genes were differentially expressed (adjusted *P* value < 0.05), 47% of which were downregulated (Fig. 3A); in *Zeb1^200H^* pituitary, 2109 genes were differentially expressed, 47% of which were downregulated (Fig. 3B). In contrast, only 44 genes were differentially expressed when comparing *Zeb1^200H^* to *Zeb1^200M^* pituitary (Fig. 3C). The fold changes observed in *Zeb1^200H^* and *Zeb1^200M^* pituitary when compared to WT pituitary were highly correlated (Pearson *R*^2^ = 0.89), and linear regression of these changes yielded a slope of 1.08 (95% confidence interval 1.08–1.09), indicating a common dysregulated program of gene expression in the two genotypes with only slightly greater magnitude of change in the *Zeb1^200M^* pituitary (Fig. 3D). Consistent with these findings, principal component analysis (PCA) of the 2000 most variable genes also indicated that the gene expression profiles of *Zeb1^200H^* and *Zeb1^200M^* pituitaries were more similar to each other than to the profile of WT pituitary (Supplementary Fig. S3A). As described below, we attribute these striking similarities between the transcriptomes of the *Zeb1^200H^* and *Zeb1^200M^* pituitaries to the miR-200a/b–ZEB1/2 DNFL.

The increased levels of ZEB1 raised the question of whether ZEB1 targets might be affected. ZEB1 acts predominantly as a transcriptional repressor, and some of its best-characterized targets are classic epithelial genes such as *Epcam* and *Cdh1* [25]. Indeed, bona fide ZEB1 targets *Epcam, Esrp1*, and *Rab25* were among the most significantly DEGs, decreasing 6–11-fold in *Zeb1^200H^* pituitary and 10–15-fold in *Zeb1^200M^* pituitary (Fig. 3E).

To systematically characterize the contribution of ZEB1 and other transcription factors to differential gene expression in *Zeb1^200M^* pituitary, we used ChEA3, a tool for analysis of enrichment of transcription-factor activity, which evaluates the overlap between user-provided gene lists and high-confidence targets for 118 transcription factors assembled from ENCODE ChIP-Seq data [53]. For each transcription factor, we plotted the gene ratio, which represents the fraction of genes in the ChIP-Seq set that were also differentially expressed in our RNA-seq data, versus the false discovery rate (FDR) (Fig. 3F). ZEB1 targets were the most overrepresented among the 1528 genes that underwent decreased expression in the *Zeb1^200M^* pituitary (36 out of 169 targets, FDR < 10^–7^). ZEB1 targets were not overrepresented among the 1751 genes that underwent increased expression in *Zeb1^200M^* pituitary, indicating that in this tissue context ZEB1 is acting primarily as a transcriptional repressor rather than activator. Instead, we found that REST targets were the most overrepresented among the genes that underwent increased expression (93 out of 337 predicted targets, FDR < 10^–19^). REST is a transcriptional repressor that silences neuronal genes in non-neuronal cells [54]. Unlike *Zeb1, Rest* mRNA was not differentially expressed in *Zeb1^200M^* pituitary, suggesting that its decreased activity was due to changes at the translational or post-translational level.

Given the central role of ZEB1 in EMT, we sought to determine whether EMT transcriptional changes were occurring in *Zeb1^200H^* and *Zeb1^200M^* pituitary. Using a core set of EMT genes derived from a meta-analysis of 18 independent studies [55], we compared genes that are commonly downregulated during EMT (epithelial or eEMT genes) and genes that are commonly upregulated during EMT (mesenchymal or mEMT genes). In both *Zeb1^200H^* and *Zeb1^200M^* pituitaries, eEMT genes were decreased, and mEMT genes were increased, with the magnitude of these effects somewhat greater in the *Zeb1^200M^* pituitary than in the *Zeb1^200H^* pituitary (Fig. 3G). Similar effects on eEMT and mEMT genes have been reported for *Zeb1^200M^* pancreatic beta-cells, indicating that these programs are largely independent of cell lineage [35, 51].

To identify additional pathways affected by elevated pituitary ZEB1, we performed gene set enrichment (GSE) analysis. The most significantly depleted gene sets were related to ribosomal protein genes and translation, whereas the most significantly enriched gene sets were related to neuronal function and synaptic activity (Fig. 3H). Increased neuronal gene expression, combined with enrichment (i.e. derepression) of REST targets, suggested a model in which miR-200a/b-mediated repression of *Zeb1* enhances silencing of neuronal genes in ectoderm-derived anterior pituitary cells. Alternatively, increased neuronal gene expression could be secondary to changes in the posterior pituitary, which consists primarily of nerve endings from neurons that originate in the hypothalamus. In the former model, the gene-expression changes would affect both mature mRNAs and pre-mRNAs; in the latter model, the gene-expression changes would only affect mature mRNAs, as the pre-mRNAs are restricted to the hypothalamus. Therefore, we aligned our pituitary RNA-seq data to intronic regions and performed differential expression analysis (Supplementary Tables S4 and S5). A strong correlation between the fold changes of exonic and intronic regions was observed for all genes (Pearson *R*^2^ = 0.77) and for neuronal genes (Pearson *R*^2^ = 0.85) when comparing *Zeb1^200M^* to WT pituitary (Fig. 3I). We also compared the lists of significantly DEGs (adjusted *P* value < .05) from analysis of exonic and intronic regions and noted a statistically significant overlap for both upregulated and downregulated genes (Fig. 3J). As expected, ZEB1 targets featured prominently in the 674 genes with decreased expression of both exons and introns; of the 36 ZEB1 targets with decreased expression in *Zeb1^200M^* pituitary (Fig. 3F), 26 also had significantly decreased intronic expression. These findings support the hypothesis that the gene expression changes in *Zeb1^200M^* pituitary are largely due to transcriptional changes in the anterior pituitary. Of note, *Zeb1* expression was increased in the *Zeb1* mutant pituitary when considering sequencing reads mapping to exons, but not when considering reads mapping to intronic regions—the expected result, given that the mutant mouse model disrupts repression of *Zeb1* by the miR-200 families, which occurs post-transcriptionally. In fact, *Zeb1* intron levels were significantly decreased 1.3-fold in *Zeb1^200M^* pituitary, suggesting that a compensatory mechanism limits *Zeb1* transcription.

Differential gene expression of pituitaries from the cohort of older females that had completed the breeding trial was highly concordant with the results from younger virgin mice (Supplementary Figs S4A–J, S5A, and S6A), indicating that the widespread changes in gene expression resulting from derepression of *Zeb1* are robust to age- and pregnancy-related differences.

### No evidence is found for *Zeb1* dysregulation in hypothalamus and ovary

As the miR-200a/b–ZEB1/2 axis has been implicated in hypothalamic control of sexual maturation and fertility [32], we also investigated gene expression across the hypothalamic-pituitary-ovarian axis in female *Zeb1^200H^* and *Zeb1^200M^* mice from both cohorts (Supplementary Tables S2 and S3). Unlike in the pituitary, *Zeb1* levels did not increase in *Zeb1* mutant hypothalamus (Supplementary Fig. S3B–S3C and S5B–S5C). Moreover, in contrast to the pituitary, a small number of genes—1 in virgin *Zeb1^200H^*, 0 in virgin *Zeb1^200M^*, 5 in older *Zeb1^200H^*, and 361 in older *Zeb1^200M^* animals—were differentially expressed in the hypothalamus (Supplementary Figs S3B–D, S5B–D, and S6B), and PCA of the 2000 most variable genes poorly separated the individual genotypes (Supplementary Figs S3A and S5A). Together, these results indicated that miR-200a/b miRNAs regulate *Zeb1* in this tissue minimally, if at all.

As in the hypothalamus, *Zeb1* levels were unchanged in the *Zeb1* mutant ovary compared to WT ovary (Supplementary Figs S3E and F and S5E and F). However, unlike in the hypothalamus, many more genes were differentially expressed in the ovary; 604 and 1319 genes were differentially expressed in virgin and older *Zeb1^200M^* animals, respectively, whereas only 76 and 21 genes were differentially expressed in virgin and older *Zeb1^200H^* animals (Supplementary Figs S3E–G and S5E–G). PCA demonstrated that *Zeb1^200M^* ovaries were the most dissimilar, whereas *Zeb1^200H^* and WT samples largely failed to separate from each other (Supplementary Figs S3A and S5A). The striking difference in *Zeb1^200M^* ovary gene expression was presumably due to the secondary effects of *Zeb1^200M^* females ovulating less frequently or not at all. Among the most downregulated genes were LH-responsive genes (e.g. *Runx2, Sfrp4*), steroidogenic enzyme genes involved in placental progesterone synthesis (e.g. *Star, Cyp11a1*), and/or corpus luteum marker genes (Supplementary Figs S3H and S5H). These findings are consistent with the ovarian dysfunction observed in *Zeb1^200M^* mice arising because of pituitary LH insufficiency and indicate that gene-expression changes in the *Zeb1^200M^* ovary accumulate over the course of the animal’s life and pregnancy history (Supplementary Fig. S6C).

### Increased *Zeb1* levels inhibit miR-200a/b expression

**Figure 4.**
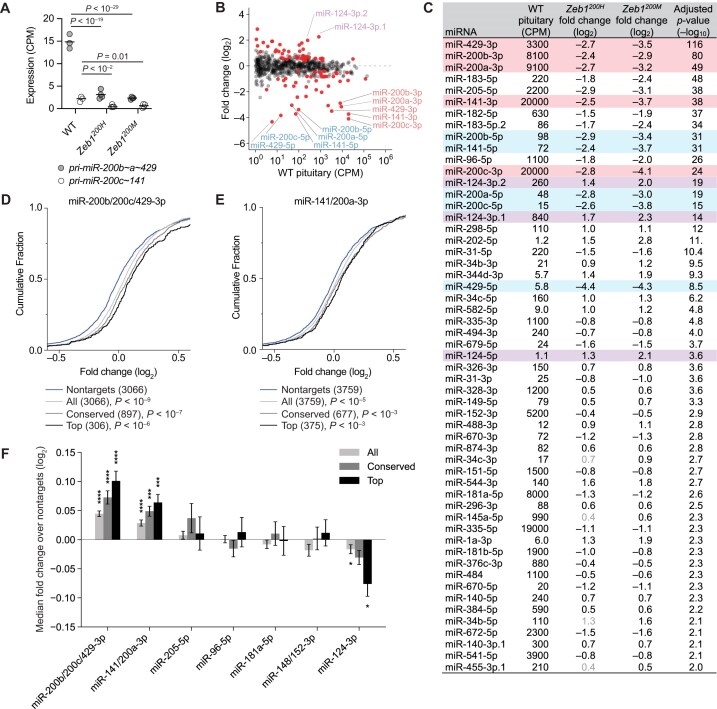
Increased ZEB1 causes decreased expression of miR-200a/b and increased accumulation of miR-200a/b targets in virgin female mice. Shown are analyses of RNA-seq and small-RNA-seq results, indicating miRNA expression and target repression in pituitary of 8–12-week-old mice staged for proestrus (*n* = 3–4 per genotype). (**A**) Influence of *Zeb1^200^* alleles on miR-200a/b family primary transcripts in the pituitary. Plotted are the CPM-mapped reads for *pri-miR-200b∼a∼429* and *pri-miR-200c∼141*, as determined by RNA-seq, in WT, *Zeb1^200H^*, and *Zeb1^200M^* pituitary (black line, mean; *n* = 3–4 per genotype). Benjamini–Hochberg adjusted *P* values, as determined by a Wald test (DESeq2), are shown for *P* values < .05. (**B, C**) The influence of *Zeb1^200M^* on mature miRNA levels in the pituitary. Plotted in panel (B) are fold changes in mean miRNA levels for *Zeb1^200M^* pituitary relative to WT pituitary, as determined by small-RNA-seq and plotted as a function of expression in WT pituitary. Each circle represents one miRNA, showing results for all miRNAs expressed above 1 CPM mapped miRNA reads (CPM). Red circles indicate differentially expressed miRNAs (adjusted *P *< .05). For each miRNA with an adjusted *P* < .01, mean WT pituitary expression, fold changes in *Zeb1^200H^* and *Zeb1^200M^* pituitary relative to WT pituitary, and adjusted *P* value for the *Zeb1^200M^* comparison are shown in panel (C). Pink denotes miRNAs of the miR-200a/b families, blue denotes passenger strands of the miR-200a/b families, and lavender denotes brain-specific miRNAs. Fold changes indicated in light gray are not statistically significant (adjusted *P* value > .05). (**D, E**) The influence of *Zeb1^200M^* on expression of miR-200b/200c/429–3p and miR-141/200a-3p predicted targets. Plotted are the cumulative distribution functions of mRNA fold changes in the *Zeb1^200M^* pituitary relative to WT pituitary for all predicted targets (light gray), conserved predicted targets (gray), top 10% predicted targets (black). The number of genes in each target set is indicated. *P* values were calculated based on a Mann–Whitney test between each target set and a set of 3′ UTR length-matched transcripts with no predicted site for each miRNA family randomly sampled at a 1:1 ratio. For simplicity, only the nontargets matched to the all targets set are plotted (blue). Selection of nontarget cohorts for each target set was repeated 21 times, and the iteration generating the median *P* value is displayed. (**F**) The influence of *Zeb1^200M^* on expression of predicted targets of differentially expressed miRNAs. Plotted are the differences between the median fold change of each set of predicted targets (all, conserved, top) and the median fold change of its matched set of nontargets. This metric of repression was calculated for 21 sampled sets of nontargets, and the mean is displayed (error bars, standard deviation). For each sampling, a *P* value was calculated as described in panels (D) and (E), and the median *P* value is displayed (*, *P *< .05; **, *P* < .01, ***, *P *< .001, ****, *P* < .0001). miR-200 family targets were excluded when calculating target repression for other miRNA families. Results for differentially expressed miRNAs with at least 1000 CPM in WT and at least 100 conserved targets (after removing miR-200 family targets) are plotted.

Given the well-known reciprocal repression that occurs between ZEB1 and miR-200a/b, we next sought to determine whether miR-200a/b levels were altered in *Zeb1^200H^* and *Zeb1^200M^* pituitary. First, we assessed expression of the primary transcripts that host the miR-200a/b miRNAs using our pituitary RNA-seq data from virgin females. In WT pituitary, *pri-miR-200b∼a∼429* levels were ∼7-fold higher than *pri-miR-200c∼141* levels (Fig. 4A). In *Zeb1^200H^* and *Zeb1^200M^* pituitaries, *pri-miR-200b∼a∼429* levels were decreased 5.3-fold and 7-fold, respectively, and *pri-miR-200c∼141* levels were decreased 4.3-fold and 3.8-fold, respectively, consistent with previous reports demonstrating that miR-200 loci are direct targets of ZEB1 [20, 26, 56] (Fig. 4A).

Small-RNA-seq of pituitaries from virgin females identified 67 and 86 miRNAs that were differentially expressed in *Zeb1^200H^* and *Zeb1^200M^* pituitary, respectively (Fig. 4B and C, Supplementary Fig. S7A, and Supplementary Table S6). The most significant of these were miR-200a/b family miRNAs, which were decreased 5.2–7.2-fold in *Zeb1^200H^* and 7.4–17.1-fold in *Zeb1^200M^* pituitary (Fig. 4B and C, pink). Consistent with the decreased levels of primary transcript, miR-200a/b passenger strands meeting the expression cut-off in WT pituitary were also substantially reduced (Fig. 4B and C, blue). In addition, several miRNAs had increased expression in *Zeb1* mutant pituitaries, including the brain-specific miRNA miR-124–3p (Fig. 4B and C, lavender). Similar to the mRNA fold changes, the miRNA fold changes observed in *Zeb1^200H^* and *Zeb1^200M^* pituitary were highly correlated (Pearson *R*^2^ = 0.94), and linear regression of these changes yielded a slope of 1.14 (95% confidence interval 1.12–1.17), indicating a common dysregulated program of gene expression in the two genotypes (Supplementary Fig. S7B).

The striking reduction in miR-200a/b levels in *Zeb1* mutant pituitaries raised the question of whether miR-200a/b targets might be affected. To answer this question, we compared fold changes of predicted miR-200a or miR-200b targets to fold changes of mRNAs lacking a miR-200a/b site. The effects on all predicted targets, conserved predicted targets, and the top 10% of predicted targets [49] were compared to UTR length-matched control sets with the expectation that higher-confidence predictions should correlate with larger effects. Indeed, we observed increased levels of both miR-200a and miR-200b targets in *Zeb1^200M^* pituitary (Fig. 4D–F) and *Zeb1^200H^* pituitary (Supplementary Fig. S7C–E). When considering the dozens of differentially expressed miRNAs in the *Zeb1^200H^* and *Zeb1^200M^* pituitary, miR-200a and miR-200b family members exerted the greatest effect on target repression, consistent with these miRNAs being both highly expressed and most significantly depleted in mutant pituitary (Fig. 4F and Supplementary Fig. S7E). Predicted targets of other downregulated miRNAs were not consistently increased after removing miR-200a/b targets, and higher-confidence predictions did not always display the greatest magnitude of change. Predicted targets of upregulated miRNAs did not decrease significantly, perhaps due to the generally lower abundance of these miRNAs. miRNA expression and target repression in pituitaries from older females that had completed the breeding trial were highly concordant with the results obtained from younger virgin mice (Supplementary Figs S8A–F and S9A–E; Supplementary Table S7).

Because *Zeb1* is one of the top predicted targets of miR-200a/b (Fig. 1B), we would expect the WT allele of *Zeb1^200H^* animals to also have increased expression, as derepression of the mutant allele causes increased ZEB1 activity, which in turn reduces miR-200a/b expression and miR-200a/b-mediated repression of WT *Zeb1* transcripts. Indeed, we found similar numbers of RNA-seq reads overlapping WT and mutant miR-200a/b sites in in *Zeb1^200H^* pituitary (Supplementary Figs S7F and S9F), indicating that the DNFL nearly fully abrogates miR-200a/b repression of WT Zeb1 allele.

Another top predicted miR-200a/b target is *Zeb2* (Fig. 1B). However, we did not observe a significant increase in *Zeb2* levels in *Zeb1* mutant pituitary from either cohort of mice (Fig. 1E and Supplementary Fig. S1E). The minor impact of the DNFL on *Zeb2* expression compared to *Zeb1* expression is consistent with the overall greater derepression of miR-200b targets compared to miR-200a targets, as *Zeb1* is predicted to be a better miR-200b target and *Zeb2* a better miR-200a target (Fig. 1B).

These results, in concert with analysis of ZEB1 transcriptional targets, indicated that disrupting the miR-200a/b–ZEB1/2 DNFL directly influences expression of hundreds of genes throughout the reproductive lifespan of the mouse. These primary effects occurred through enhanced transcriptional repression by ZEB1 and reduced post-transcriptional repression by miR-200a/b. When adding on downstream, secondary effects, expression of >2000 genes was affected.

## Discussion

In this study, we characterize the physiological consequences of derepressing a single miRNA target in mice. Loss of miR-200a/b regulation of *Zeb1* caused anovulatory infertility and reduced LH expression in female mice. Some previous studies have also mutated miRNA complementary sites to investigate the phenotypic consequences of disrupting miRNA regulation of a single target [57–73]. In mice, this approach has revealed that miRNA regulation of a single gene can improve synaptic plasticity and memory [70], alter bone development [71], and affect various phenomenon in immune cells, including response to infection [62], proliferation of thymocytes [65], and derepression of cytidine deaminase activity [58, 59]. With respect to evolutionary fitness of the organism, the fertility phenotypes we observed upon disruption of miR-200a/b repression of murine *Zeb1* are the most severe phenotypes observed for reduced miRNA regulation of a single mammalian mRNA and are among the most severe observed for mutation of a single mammalian 3′ UTR.

**Figure 5.**
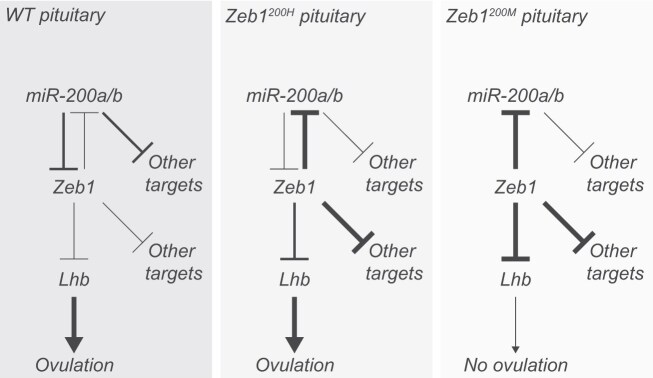
Increased ZEB1 activity in *Zeb1^200H^* and *Zeb1^200M^* pituitary represses miR-200a/b miRNAs, *Lhb*, and other ZEB1 targets. Line thicknesses indicate relative strengths of repression. Only *Zeb1^200M^* mice have enough ZEB1 activity to impair ovulation.

The fertility defects of mice with this miR-200a/b-resistant *Zeb1* 3′ UTR mirrored the fertility defects previously reported for miR-200b/429 double-knockout mice [34] and for mice with gonadotrope-specific overexpression of ZEB1 [34]. Our results, combined with the results of these previous studies, converge on a model in which derepression of *Zeb1* is the driving cause of infertility associated with loss of miR-200b/429, and more generally, that miR-200a/b limits *Zeb1* expression in pituitary gonadotrope cells to promote *Lhb* expression and ovulation (Fig. 5).

Despite the severity of the *Zeb1*^200M^ phenotype, the extent to which *Zeb1* repression accounts for the biological effects of the miR-200a/b miRNA superfamily is unclear. Assessing this question would require more information on the phenotype of a complete loss of the miR-200a/b superfamily, which could be even more severe than the loss of female fertility observed upon loss of miR-200b/429.

### Primary defect occurs in the pituitary

Although *Zeb1^200M^* mice are resistant to miR-200a/b in all cells and tissues, derepression of *Zeb1* within the hypothalamic-pituitary-ovarian axis appears to be restricted to the pituitary. Increased *Zeb1* expression was detected in *Zeb1^200M^* pituitary but not in *Zeb1^200M^* hypothalamus or ovary (Fig. 1E and 3A). Likewise, increased repression of well-established ZEB1 targets, such as *Epcam, Esrp1*, and the miR-200a/b family primary transcripts, was observed exclusively in pituitary (Fig. 3E and 4A). These results are consistent with previous work demonstrating that ZEB1 protein is increased in the pituitary but not the hypothalamus of miR-200b/429 double-knockout mice and that forced expression of ZEB1 specifically in gonadotrope cells recapitulates the anovulation observed in miR-200b/429 double-knockout mice [34].

On the other hand, we found no evidence for a role of the miR-200a/b–Zeb1 DNFL in hypothalamus. Hypothalamic neurons produce GnRH, which stimulates expression and secretion of LH and FSH in the anterior pituitary. Complete loss of miRNAs in hypothalamic neurons blunts GnRH expression and sexual maturation, which ultimately results in both male and female sterility [32]. Although miR-200a/b regulation of *Zeb1* is reported to mediate hypothalamic control of GnRH expression [32], our miR-200a/b-resistant *Zeb1* mice had normal sexual maturation; male *Zeb1^200M^* mice were fertile, and female *Zeb1^200M^* mice had normal estrous cycles (Fig. 2D and Supplementary Fig. S2B). In our study, *Zeb1^200M^* ovaries were smaller than WT ovaries, but we speculate that this size difference reflects a lack of corpus lutea (Supplementary Fig. S2C). Thus, miR-200a/b regulation of *Zeb1* is not required for proper hypothalamic control of GnRH expression.

### *Zeb1* levels are tightly regulated

The 1.4–1.5-fold increase in *Zeb1* transcripts in *Zeb1^200M^* pituitary was modest but in line with previous observations. Similar *Zeb1* fold changes are reported in cancer cell lines with reduced levels of miR-200a/b and in *Zeb1^200M^* pancreatic islets [21–23, 35, 51]. As in the pituitary, these fold changes have functional consequences. For example, the ∼1.5-fold increase in *Zeb1* mRNA in pancreatic islets, accompanied by a ∼1.3-fold increase in ZEB1 protein, is sufficient to promote EMT, tumor invasion, and increased metastasis in two different autochthonous cancer models [35].

How does the magnitude of *Zeb1* derepression compare to that of other miRNA targets? In general, median fold changes of derepression in miRNA knockout and knockdown experiments range from ∼1.1–1.3-fold for sets of top predicted targets, with some of the variation in observed fold changes presumably due to differences in miRNA abundance and/or incomplete depletion of the miRNA [39, 49]. Of course, most mRNAs that are targeted by one miRNA are also targeted by other miRNAs, and thus the overall effect of miRNAs on protein output can be much higher than 1.3-fold [74]. In *Zeb1^200M^* pituitary, which has ∼10-fold lower expression of miR-200a and miR-200b families than WT pituitary (Fig. 4B), median fold changes of derepression were ∼1.1-fold for top predicted miR-200a/b targets (Fig. 4F). In that regard, the 1.4–1.5-fold increase in *Zeb1* was well above the median. Indeed, *Zeb1* ranked among the 20% most derepressed targets within our set of predicted top targets. Furthermore, RNA-seq reads mapped to introns revealed downregulation of *Zeb1* at the transcriptional level in *Zeb1^200M^* pituitary from virgin female mice (Supplementary Table S4), indicating that additional layers of transcriptional control may blunt post-transcriptional derepression. We also found that ZEB1 protein levels appeared to be further increased in miR-200a/b-resistant *Zeb1* pituitary (Fig. 1F and G), possibly due to derepression of translation.

Another way we might have underestimated the magnitude of derepression is if miR-200a/b regulation of *Zeb1* occurs in only some of the ∼12 distinct pituitary cell types. However, our data suggest that increased ZEB1 activity was likely occurring throughout the pituitary. In single-nuclei RNA-seq from WT female mouse pituitary, *Zeb1* is expressed at similar levels across the six main hormone-producing cell types, which account for >80% of all cells in the pituitary [75]. Likewise, ZEB1 targets *Epcam, Esrp1*, and *Rab25*, which decreased 10–15-fold in *Zeb1^200M^* pituitary (Fig. 3E), are each expressed at similar levels across the hormone-producing cell types [75]. Because these ZEB1 targets are broadly expressed at similar levels, the striking fold changes we observed in *Zeb1^200M^* pituitary were only possible if ZEB1 activity increased in most pituitary cells.

### *Lhb* is unusually sensitive to ZEB1 dosage

The gene expression profiles of *Zeb1^200H^* and *Zeb1^200M^* pituitary were highly correlated, with only slightly larger-magnitude fold changes in *Zeb1^200M^* pituitary when each was compared to WT pituitary (Fig. 3D and Supplementary Fig. S4D). For instance, in *Zeb1^200H^* pituitary, *Zeb1* expression increased to ∼95% of the level observed in *Zeb1^200M^* pituitary (Fig. 1E), and several ZEB1 targets, including *Esrp1, Rab25*, and the two miR-200a/b primary transcripts, were not differentially expressed when comparing *Zeb1^200M^* pituitary to *Zeb1^200H^* pituitary (Supplementary Table S2). We attribute these transcriptomic similarities between the *Zeb1^200H^* and the *Zeb1^200M^* pituitary to the DNFL, in which derepression of the mutant *Zeb1^200H^* allele causes increased ZEB1 activity, which reduces *Mir200* transcription and miR-200a/b-mediated repression of mRNA from the WT *Zeb1^200H^* allele, such that the combined level of both *Zeb1* alleles of the *Zeb1^200H^* pituitary resembles that of the *Zeb1^200M^*<?brk ?> pituitary.

Despite the high degree of overlap between *Zeb1^200H^* and *Zeb1^200M^* pituitary at the molecular level, only *Zeb1^200M^* mice had ovulation defects and impaired fertility (Fig. 2A and G). Two lines of evidence indicate that this phenotypic difference was due to differences in LH expression observed between *Zeb1^200H^* and *Zeb1^200M^* mice. First, administration of hormones used for superovulation restores ovulation in miR-200b/429 double-knockout mice [34]. Second, *Lhb* transcripts, *Lhb* intron-mapping reads, and circulating LH protein were each decreased ~50% less in *Zeb1^200H^* mice than in *Zeb1^200M^* mice (Fig. 2H and J; Supplementary Tables S2 and S3). In fact, *Lhb* was one of only 44 genes that was differentially expressed in virgin females when comparing *Zeb1^200M^* pituitary to *Zeb1^200H^* pituitary (Supplementary Table S2). This proposed mechanism implies that *Lhb* transcription is exquisitely sensitive to the small increase in *Zeb1* levels observed between *Zeb1^200H^* and *Zeb1^200M^* pituitary. The average differences in *Zeb1* mRNA levels, which we measured to be 5%, and in ZEB1 protein levels, which we measured to be 13%, were so small that neither achieved statistical significance when comparing *Zeb1^200H^* and *Zeb1^200M^* pituitary. Thus, we infer that some small and likely difficult-to-quantify increase in ZEB1 between *Zeb1^200H^* and *Zeb1^200M^* pituitary must somehow explain the sizable *Lhb* decrease and fertility phenotype. One possibility is that transcription of *Lhb* is ultra-sensitive to the level of ZEB1, perhaps through highly cooperative ZEB1 binding to multiple sites in the *Lhb* promoter [34, 52], such that a small increase in ZEB1 when comparing *Zeb1^200M^* to *Zeb1^200H^* pituitary is sufficient to reduce *Lhb* transcription more than two-fold. Alternatively, the genes that undergo differential expression in *Zeb1^200M^* pituitary compared to *Zeb1^200H^* pituitary might include multiple regulators that cooperatively converge with ZEB1 to reduce *Lhb* transcription.

### Importance of the miR-200a/b–ZEB1/2 DNFL

Our study of miR-200a/b-resistant *Zeb1* mice, together with the previously reported findings of miR-200b/429 double-knockout mice [34], demonstrates that miR-200a/b-mediated repression of *Zeb1* increases pituitary *Lhb* expression and circulating LH protein to physiological levels that support ovulation and female fertility (Fig. 5). This phenotype observed in miR-200a/b-resistant *Zeb1* mice differs from the phenotype observed with complete loss of *Lhb* expression, which causes hypogonadism and infertility in both male and female mice [4], indicating that the small amount of circulating LH detected in female miR-200a/b-resistant *Zeb1* mice is sufficient for gonadogenesis. Alternatively, because our measurements of *Lhb* transcripts and circulating LH protein were all taken from adult female mice during proestrus, when *Lhb* transcription and LH secretion are maximal, the molecular changes we observed might be limited to adult mice or to one or more stages of the estrus cycle. Age- and stage-resolved measurements of *Zeb1*, miR-200a/b, and *Lhb* in WT and *Zeb1^200M^* pituitary gonadotropes will be necessary to determine whether the miR-200a/b–ZEB1/2 DNFL acts continuously to suppress ZEB1 activity and promote *Lhb* transcription or serves a more dynamic purpose, for example, as an ultrasensitive, inducible switch for *Lhb* expression.

Like LH secretion, *Lhb* transcription is induced during proestrus by changes in the frequency of hypothalamic GnRH pulses [76–79]. This GnRH signal is transduced by EGR1, SF-1, and PITX1, transcriptional activators that bind directly to the *Lhb* promoter [80–83]. Less is known about the repressive factors that might restrict the duration and amplitude of GnRH-induced *Lhb* transcription. Our study and prior findings in mice and pituitary cell lines indicate that ZEB1 may play such a role: ZEB1 binds the *Lhb* promoter [34, 52], and increased expression of ZEB1 blocks GnRH- or EGR1-stimulated *Lhb* expression in the murine LbetaT2 gonadotrope cell line [52] and potently suppresses *Lhb in vivo* (Fig. 2J) [34], whereas transient knockdown of *Zeb1* in LbetaT2 cells increases *Lhb* levels [34].

Although increased ZEB1 activity in pituitary gonadotropes impairs female fertility, decreased ZEB1 activity appears to be better tolerated. Female mice with gonadotrope-specific deletion of *Zeb1* are fertile and have normal levels of pituitary *Lhb* and circulating LH, at least during diestrus [52]. Further investigation is needed to determine the impact of gonadotrope-specific ZEB1 deficiency on *Lhb* expression during other stages of the estrus cycle and after treatment with a GnRH agonist. Because constitutive overexpression of *Lhb* in gonadotropes causes anovulatory infertility and ovarian cysts and tumors [84], we speculate that mammals might have redundant mechanisms to limit *Lhb* expression and secretion.

Is ZEB1 regulation of *Lhb* conserved? To date, the effects of physiological levels of ZEB1 on *Lhb* expression have been reported only in mice and murine cell lines. When examining the effects of ZEB1 ectopic overexpression, a partial fragment of the human *LHB* promoter is detectably repressed, although the magnitude of repression is less than that observed with an orthologous fragment of the mouse *Lhb* promoter [52]. This differential sensitivity can be at least partly attributed to two E-box motifs that are present in the mouse *Lhb* promoter but not in the human *LHB* promoter. The distal motif is also found in ungulates (pigs, cows) and elephants, and the proximal motif is found in most placental mammals surveyed, except for humans and chimpanzees (Supplementary Fig. S10A). For both mouse and human promoter fragments, some of the ZEB1 repression appears to be independent of canonical E-box motifs, suggesting either non-canonical binding or indirect effects of ZEB1 [52]. It is also worth noting that the promoter fragments used in this study end shortly after the transcription start site (TSS) and thus do not include an additional E-box motif downstream of the TSS (+18 bp), which is the most deeply conserved E-box motif within 500 bp of the TSS in either direction (Supplementary Fig. S10A). The importance of this motif in ZEB1-mediated repression of *Lhb* awaits further investigation.

Although *Lhb* is restricted to placental mammals, orthologues of both miR-200 and ZEB1/2 have existed for >600 million years, i.e. since the last common ancestor of Bilateria. Even so, the miR-200–ZEB DNFL appears to be a more recent innovation (Supplementary Fig. S10B). Multiz alignments of the *Zeb1* and *Zeb2* 3′ UTRs indicate that miR-200a/b repression of *Zeb1* and *Zeb2* likely originated with vertebrates (Fig. 1C and Supplementary Fig. S1C). Multiz alignments of the *Mir200b∼a∼429* and *Mir200c∼141* promoters suggest that ZEB1/2 repression of *Mir200b∼a∼429* also likely originated with vertebrates, whereas ZEB1/2 repression of the mammalian-specific *Mir200c∼141* cluster likely originated with placental mammals (Supplementary Fig. S10C). While most functional studies of the miR-200a/b–ZEB1/2 DNFL have focused on mice and human cell lines, one group showed in zebrafish embryos that *zeb1b* overexpression causes a ∼2-fold decrease in miR-200a/b miRNAs and morpholino-based knockdown of *zeb1a/b* causes a ∼2-fold increase in miR-200a/b miRNAs [85]. Nevertheless, miR-200a/b knockdown did not recapitulate the developmental cell adhesion defects observed with overexpression of *zeb1b*, indicating that the miR-200a/b–ZEB1/2 DNFL is likely not required for the developmental function ascribed to *zeb1a/b*. Understanding how the miR-200a/b–ZEB1/2 DNFL functions in zebrafish and other animals may reveal new and common principles about this ancient gene regulatory circuit.

## Supplementary Material

gkaf1357_Supplemental_Files

## Data Availability

Sequencing datasets generated in this study have been deposited in the GEO under accession numbers GSE279446 and GSE279447.

## Notes

Present address: Pfizer Research and Development, Pearl River, NY 10965, United States
