# Supplementary material for: Derepression of a single microRNA target causes female infertility in mice
Source: Nucleic Acids Res. 2026 Jan 14;54(2):gkaf1357. doi: 10.1093/nar/gkaf1357 (PMC12802903; doi:10.1093/nar/gkaf1357)
Supplement: gkaf1357_Supplemental_Files [file gkaf1357_supplemental_files.zip › Supplementary_Data.pdf]

**Offspring of *Zeb1 6B6*^{200H} x *Zeb1 6B6*^{200H} Cross**			
	Zeb1 6B6$^{+/+}$	*Zeb1 6B6*^{200H}	*Zeb1 6B6*^{200M}
Observed (P21)	58	108	42
% observed	28	52	20
% expected	25	50	25

Shown are results of crossing heterozygous mice on a C57Bl/6J background (>15 backcrosses) derived from targeted ESC clone 6B6, which has mutations in all 9 miR-200a/b sites in the *Zeb1* 3′ UTR. Statistical significance was determined by X^2 test (P = 0.25).

**Offspring of *Zeb1 3C5*^{200H} x *Zeb1 3C5*^{200H} Cross**			
	Zeb1 3C5$^{+/+}$	*Zeb1 3C5*^{200H}	*Zeb1 3C5*^{200M}
Observed (P21)	35	69	26
% observed	27	53	20
% expected	25	50	25

Shown are results of crossing heterozygous mice on a C57Bl/6J background (>15 backcrosses) derived from targeted ESC clone 3C5, which has mutations in all 9 miR-200a/b sites in the *Zeb1* 3′ UTR. Statistical significance was determined by X^2 test (P = 0.42).

**Offspring of *Zeb2 3E3*^{200H} x *Zeb2 3E3*^{200H} Cross**			
	Zeb2 3E3$^{+/+}$	*Zeb2 3E3*^{200H}	*Zeb2 3E3*^{200M}
Observed (P21)	10	18	10
% observed	26	47	26
% expected	25	50	25

Shown are results of crossing heterozygous mice on a C57Bl/6J background (>10 backcrosses) derived from targeted ESC clone 3E3, which has mutations in the first 10 miR-200a/b sites in the *Zeb2* 3′ UTR. Statistical significance was determined by X^2 test (P = 0.95).

Supplementary Table S1. Viability of mutant mice in this study, related to Figure 1

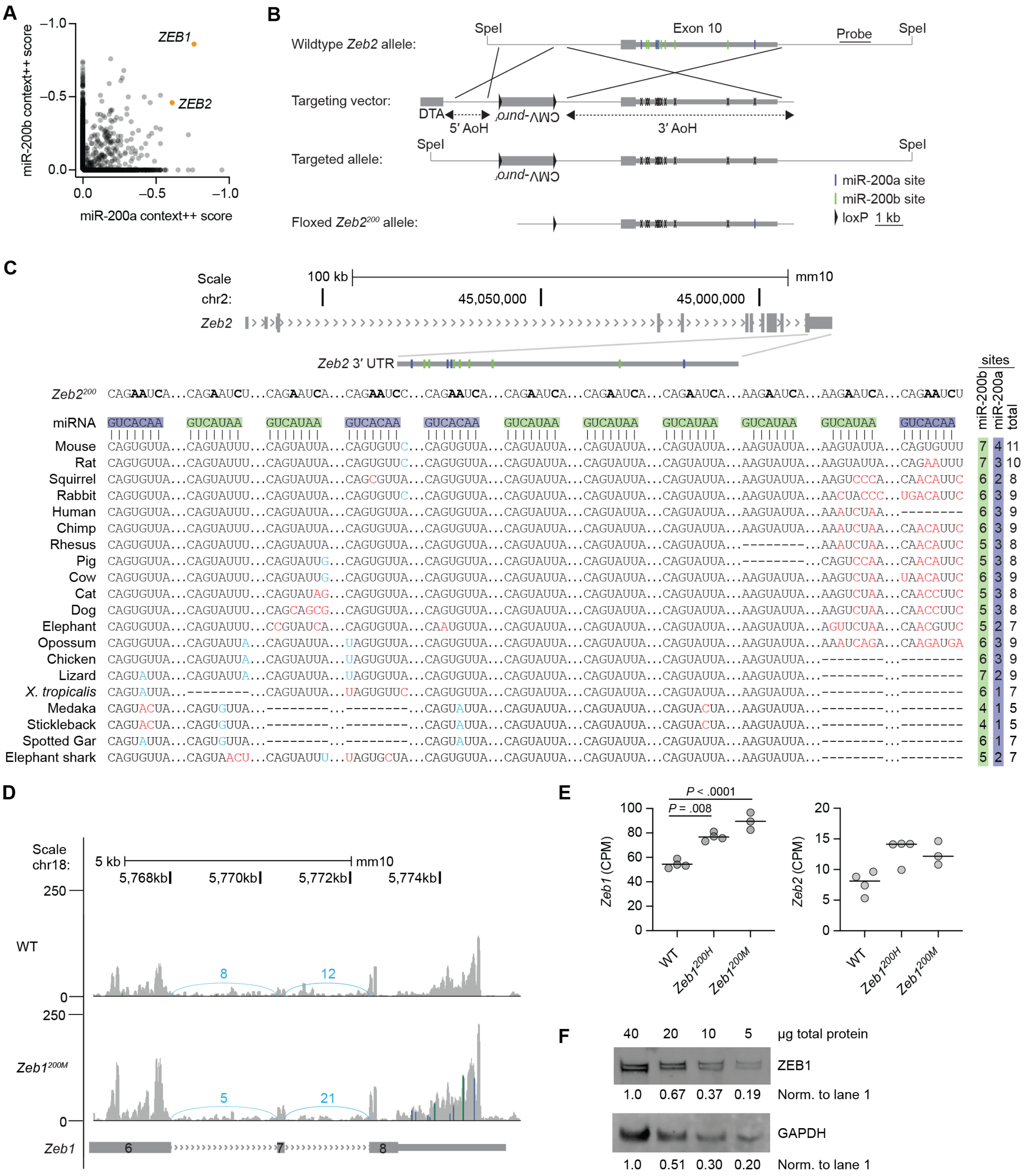

Supplemental Figure S1. *Zeb1* is a top target of the miR-200 superfamily, related to Figure 1. (A) Predicted targets of miR-200b/c/429 and miR-141/200a in humans. Otherwise, as in Figure 1B. (B) *Zeb2* gene targeting strategy. Shown between the wild-type exon 10 and the recombinant alleles is the vector used to insert 2–3 point mutations into each miR-200a/b site (gray boxes, exons; green lines, miR-200b sites; blue lines, miR-200a sites; X, mutant sites; filled triangles, loxP sites; CMV-*puro*ʳ,

puromycin-resistance cassette; DTA, diphtheria toxin A; AoH, arm of homology). Screening of ES cell clones was performed by long-range PCR as well as Southern blot of SpeI-digested genomic DNA with a 3′ probe downstream of exon 10. (C) Organization of the murine *Zeb2* locus. The *Zeb2* gene model (gray boxes, exons; > > >, introns) is depicted with an inset of the 3′ UTR indicating the location of the binding sites for miR-200b (green boxes) and miR-200a (blue boxes). Otherwise, as in Figure 1C. (D) The minimal influence of miRNA binding site mutations on *Zeb1* splicing and 3′ end formation. RNA-seq coverage tracks for wild-type and $Zeb1^{200M}$ pituitary from 8–12-week-old female mice (*y*-axis, average number of reads per genomic window) derived from libraries with similar sequencing depths (~40 million reads for each library) and visualized in IGV are shown above the last three exons of the *Zeb1* gene model (gray boxes, exons; > > >, introns). Single nucleotide mismatches to the reference sequence are indicated in green (adenine) and blue (cytosine) and reflect successful mutagenesis of miR-200a/b binding sites. Splice junctions are represented by arcs with the numbers above each arc reflecting unique splice junction mapping reads. (E) Influence of $Zeb1^{200}$ alleles on *Zeb1* and *Zeb2* expression in the pituitary of 28–36-week-old female mice. Otherwise, as in Figure 1E. (F) Dynamic range of ZEB1 and GAPDH detection. Shown is a western blot probing total protein from the pituitary of an 8–12-week-old $Zeb1^{200H}$ female mouse. The amount of protein loaded in each lane is indicated above, and the band intensities for ZEB1 and GAPDH are indicated below, relative to the left-most lane.

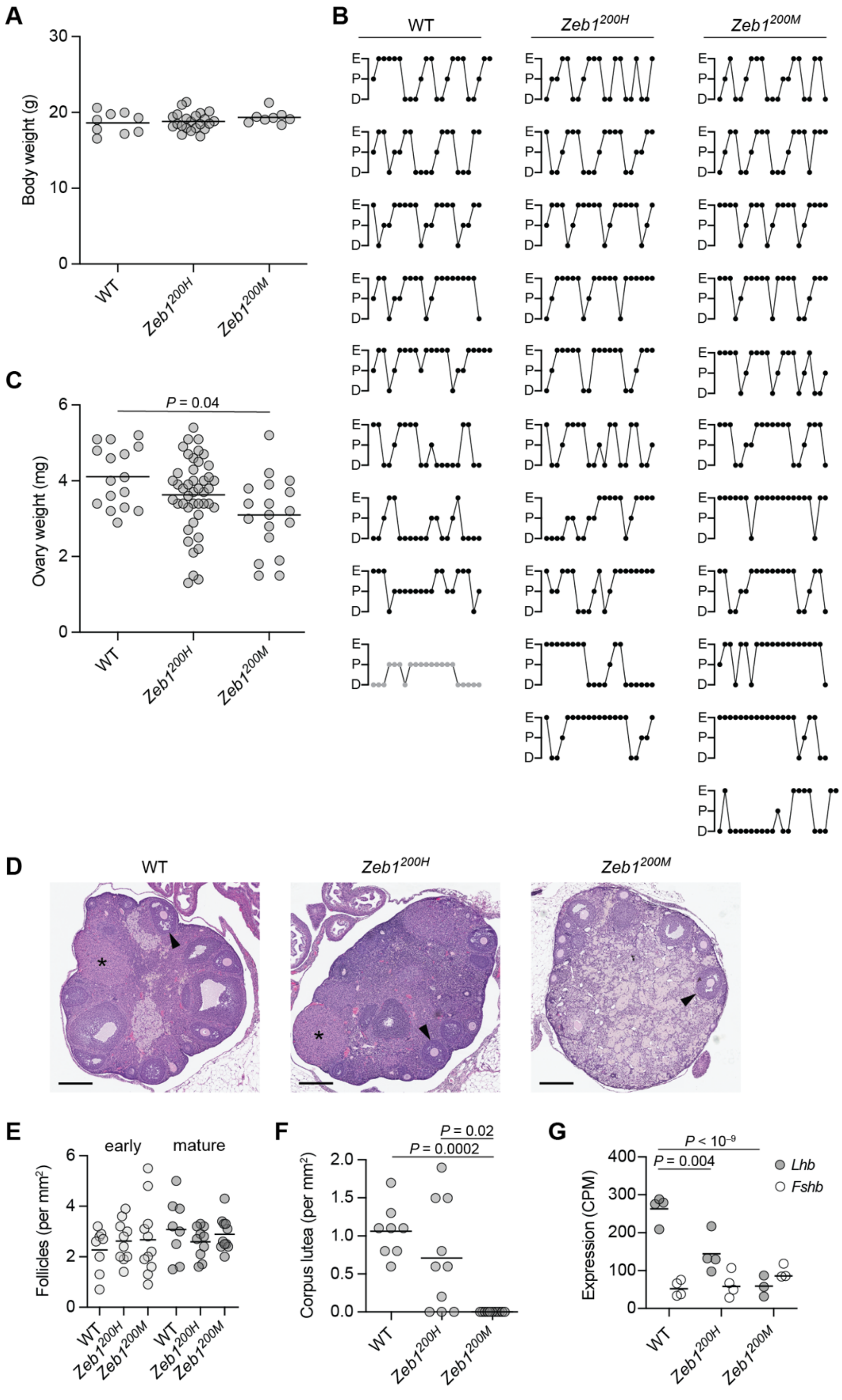

Supplemental Figure S2. Female *Zeb1*^{200M} mice have decreased fertility, related to Figure 2. (A) Influence of *Zeb1*^{200} allele on body weight. Plotted are weights (in grams) for WT, *Zeb1*^{200H}, and *Zeb1*^{200M} female mice at 8–12 weeks of age (black line, mean; n = 9–22 mice per genotype). No statistically significant changes (adjusted P value <

0.05) were detected (ANOVA with Kruskal–Wallis test). (B) Influence of *Zeb1*^{200} alleles on estrus cycle length and stage. Plotted are consecutive measurements of estrus cycle stage, as determined by vaginal cytology, beginning at 10 weeks of age for 9 WT, 10 *Zeb1*^{200H}, and 11 *Zeb1*^{200M} female mice. Each dot represents one day (n = 21–23 days per animal; E, estrus; P, proestrus; D, diestrus). Of the 30 mice in our cohort, 29 completed at least one estrus cycle and were included in the subsequent breeding trial; one WT mouse (gray dots) never cycled and was excluded from further studies. (C) Influence of *Zeb1*^{200} allele on ovary weight. Plotted are weights for WT, *Zeb1*^{200H}, and *Zeb1*^{200M} ovaries at 8–12 weeks of age, staged for proestrus (black line, mean; n = 18–44 ovaries per genotype from 9–22 mice). Only statistically significant changes (adjusted P value < 0.05) for all pairwise comparisons are indicated (linear mixed-effects model ANOVA, with Tukey's multiple comparison test). (D–F) Influence of *Zeb1*^{200} alleles on ovarian folliculogenesis. Shown in (D) are hematoxylin and eosin stained sections of ovary from 27–40-week-old WT, *Zeb1*^{200H}, and *Zeb1*^{200M} female mice at the conclusion of the breeding trial (arrowhead, secondary/mature follicle; asterisk, corpus luteum; scale bar, 300 μm). The number of early/primary and mature/secondary follicles per mm^2 ovary section and the number of corpus luteum per mm^2 ovary section are plotted in (E) and (F), respectively, for the indicated genotypes (black line, mean; n = 7–10 mice per genotype). Only statistically significant changes (adjusted P value < 0.05) for all pairwise comparisons are indicated (ANOVA with Kruskal–Wallis test). (G) Influence of *Zeb1*^{200} alleles on *Lhb* and *Fshb* expression in the pituitary. Plotted are the counts per million mapped reads (CPM) for *Lhb* and *Fshb*, as determined by RNA sequencing, in the pituitary of 28–36-week-old WT, *Zeb1*^{200H}, and *Zeb1*^{200M} female mice at the conclusion of the breeding trial. Otherwise, as in Fig. 2J.

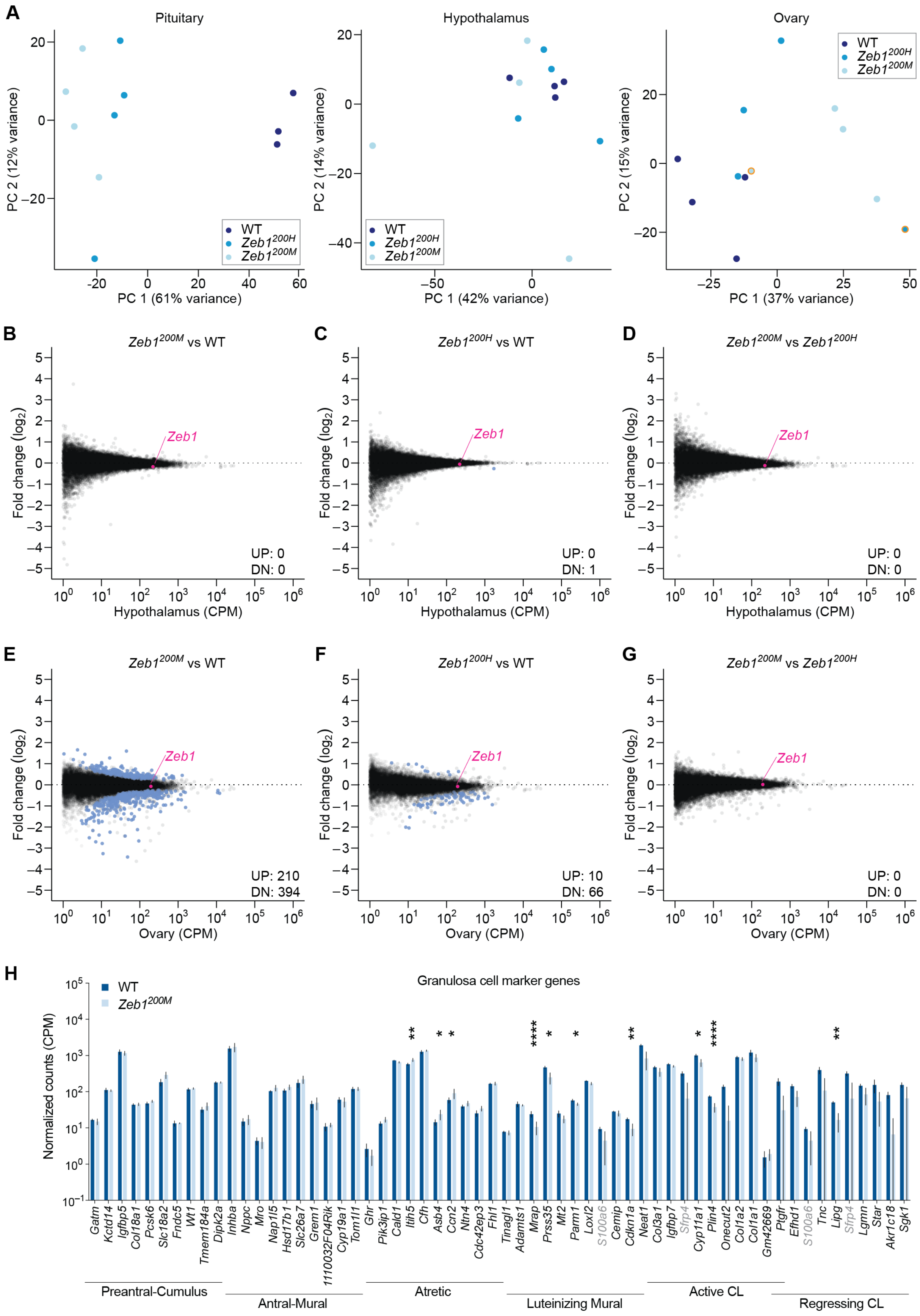
A
Pituitary
Hypothalamus
Ovary
PC 1 (61% variance)
PC 2 (12% variance)
PC 1 (42% variance)
PC 2 (14% variance)
PC 1 (37% variance)
PC 2 (15% variance)
WT
Zeb1200H
Zeb1200M
B
Zeb1200M vs WT
C
Zeb1200H vs WT
D
Zeb1200M vs Zeb1200H
Fold change (log2)
Hypothalamus (CPM)
Zeb1
UP: 0
DN: 0
UP: 0
DN: 1
UP: 0
DN: 0
E
Zeb1200M vs WT
F
Zeb1200H vs WT
G
Zeb1200M vs Zeb1200H
Ovary (CPM)
UP: 210
DN: 394
UP: 10
DN: 66
UP: 0
DN: 0
H
Granulosa cell marker genes
WT
Zeb1200M
Normalized counts (CPM)
Gatm
Kctd14
Igfbp5
Col18a1
Pcsk6
Slc18a2
Fndc5
Wt1
Tmem184a
Dlpk2a
Inhba
Nppc
Mro
Nap1l5
Hsd17b1
Slc26a7
Grem1
1110032F04Rik
Cyp19a1
Tom1l1
Ghr
Pik3ip1
Cald1
Itih5
Cfh
Asb4
Ccn2
Ntn4
Cdc42ep3
Fhl1
Tinagl1
Adamts1
Mrap
Prss35
Mt2
Parm1
Loxl2
S100a6
Cemip
Cdkn1a
Neat1
Col3a1
Igfbp7
Sfrp4
Cyp11a1
Plin4
Onecut2
Col1a2
Col1a1
Gm42669
Ptgfr
Efhd1
S100a6
Tnc
Lipg
Sfrp4
Lgmn
Star
Akr1c18
Sgk1
Preantral-Cumulus
Antral-Mural
Atretic
Luteinizing Mural
Active CL
Regressing CL

Supplemental Figure S3. Widespread gene expression changes induced by modest derepression of *Zeb1* in virgin female mice, related to Figure 3. Shown are analyses of RNA-seq results from WT and *Zeb1*^{200} mutant tissues from 8–12-week-old mice staged for proestrus (*n* = 3–4 per genotype). (A) Principal component analysis. Plotted are the first two principal components (PC1, PC2) for pituitary (left), hypothalamus (center), and ovary (right) based on analysis of the 2000 most variable genes derived from RNA sequencing libraries. Each circle represents a unique sample/library and each genotype is represented by a different color (WT, dark blue; *Zeb1*^{200H}, blue; *Zeb1*^{200M}, light blue). In the ovary analysis (right), two samples are marked with orange outlines: one *Zeb1*^{200H} sample from a mouse with no corpus lutea detected in the contralateral ovary and one *Zeb1*^{200M} sample from a mouse with corpus lutea detected in the contralateral ovary. (B–D) The influence of *Zeb1*^{200} alleles on RNA levels in the hypothalamus; otherwise, as in Figure 3A–C. (E–G) The influence of *Zeb1*^{200} alleles on RNA levels in the ovary; otherwise, as in Figure 3A–C. (H) The influence of *Zeb1*^{200M} on expression of granulosa cell marker genes in the ovary. Plotted are the mean counts per million mapped reads (CPM), as determined by RNA sequencing, for 60 genes previously attributed to six different granulosa cell types (Morris et al. 2022) in WT and *Zeb1*^{200M} ovary (WT, dark blue; *Zeb1*^{200M}, light blue; CL, corpus lutea; error bars, standard deviation; *n* = 3–4 per genotype). Benjamini–Hochberg adjusted *p* values, as determined by a Wald test (DESeq2), are indicated (*, $P < 0.05$; **, $P < 0.01$, ***, $P < 0.001$, ****, $P < 0.0001$). Two genes, *S100a6* and *Sfrp4*, indicated by gray text are attributed to two different granulosa cell types.

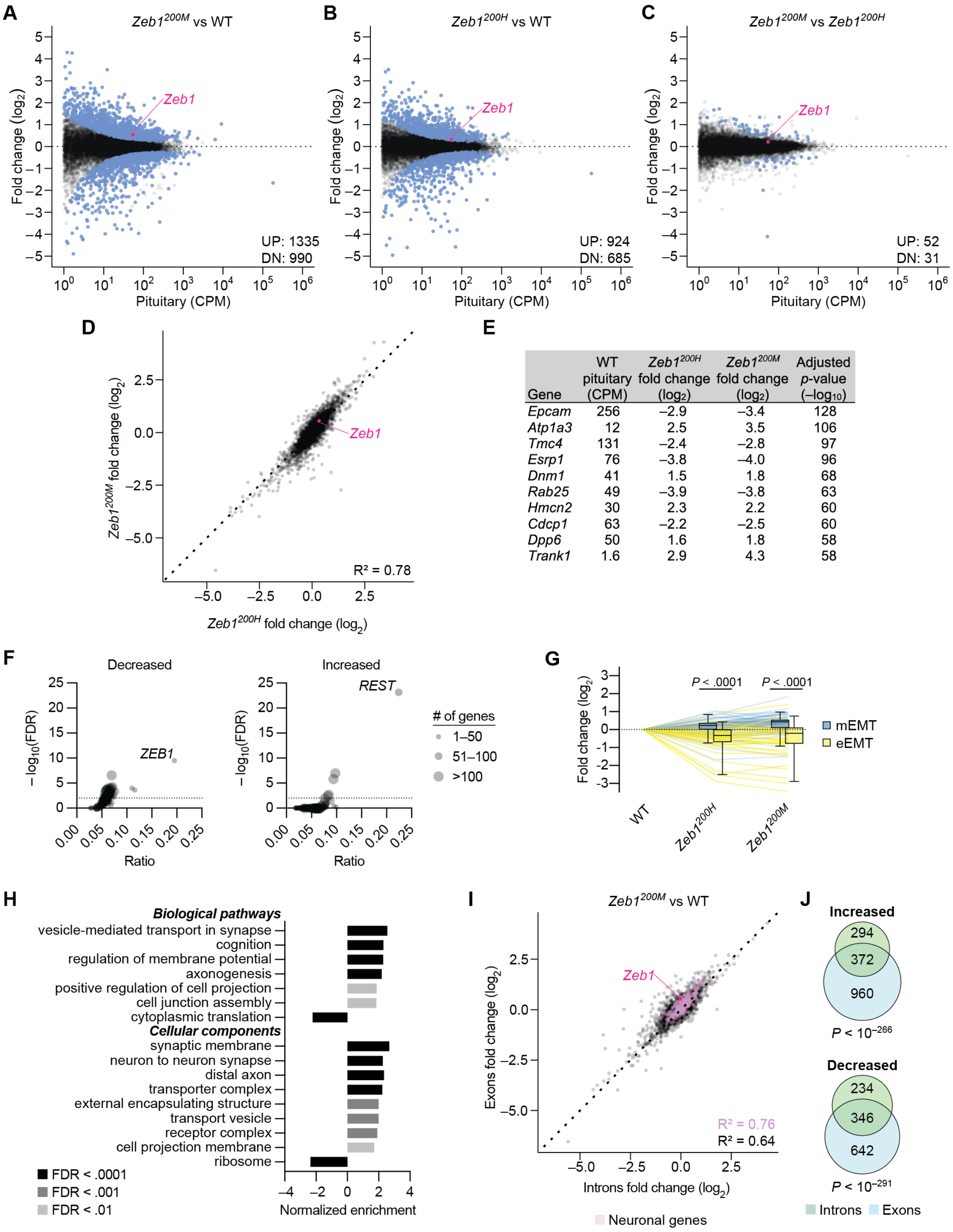

Supplemental Figure S4. Widespread gene expression changes induced by modest derepression of *Zeb1* in older female mice, related to Figure 3. Shown are analyses of RNA-seq results from pituitary of 28–36-week-old WT, *Zeb1*^{200H}, and

Zeb1^{200M} female mice staged for proestrus at the conclusion of the breeding trial (*n* = 3–4 per genotype). Otherwise, this panel is as in Figure 3A–J**.**

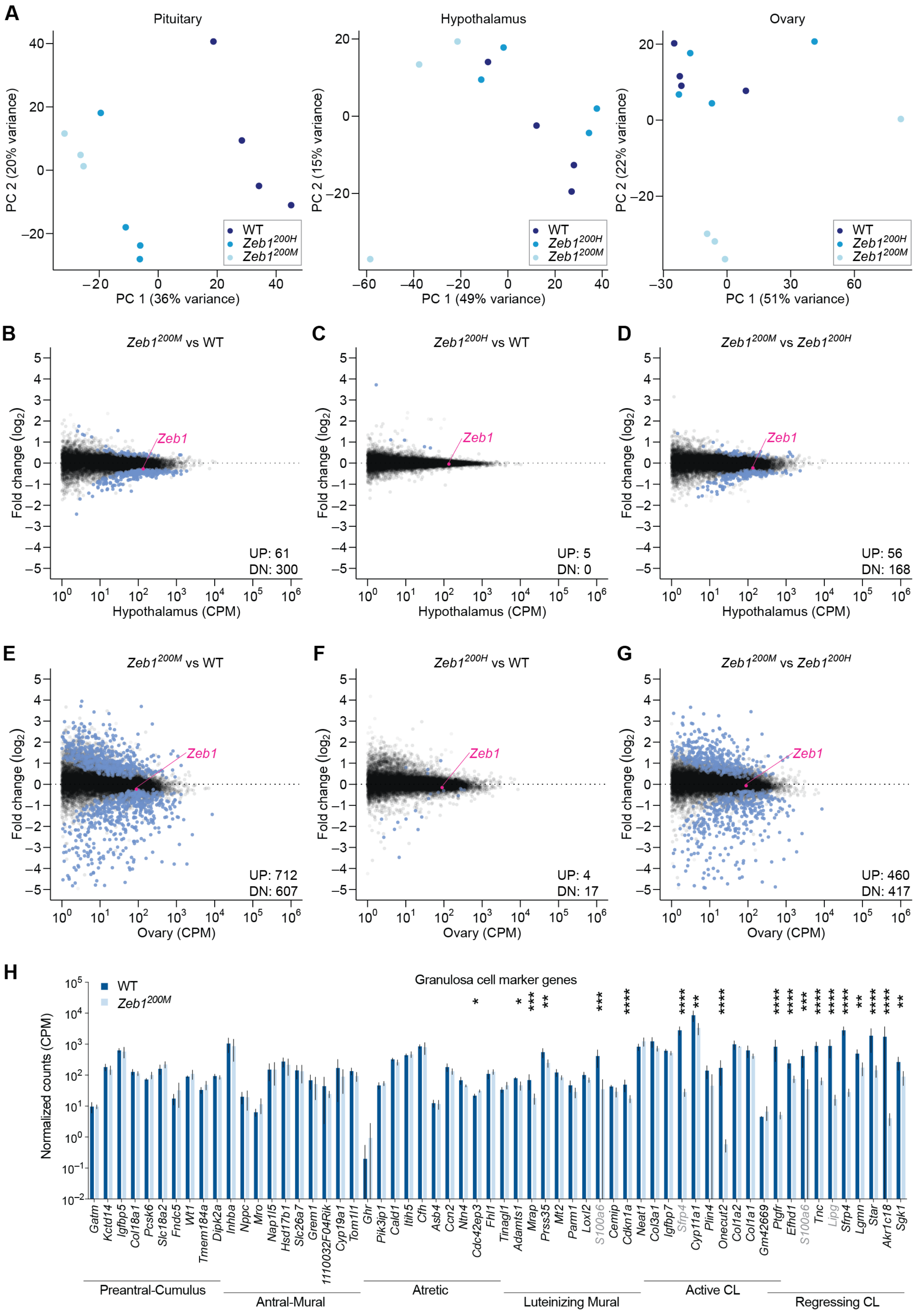
A
Pituitary
Hypothalamus
Ovary
PC 2 (20% variance)
PC 1 (36% variance)
PC 2 (15% variance)
PC 1 (49% variance)
PC 2 (22% variance)
PC 1 (51% variance)
WT
Zeb1200H
Zeb1200M
B
Zeb1200M vs WT
C
Zeb1200H vs WT
D
Zeb1200M vs Zeb1200H
Fold change (log2)
Zeb1
UP: 61
DN: 300
UP: 5
DN: 0
UP: 56
DN: 168
Hypothalamus (CPM)
E
Zeb1200M vs WT
F
Zeb1200H vs WT
G
Zeb1200M vs Zeb1200H
UP: 712
DN: 607
UP: 4
DN: 17
UP: 460
DN: 417
Ovary (CPM)
H
Granulosa cell marker genes
WT
Zeb1200M
Normalized counts (CPM)
Gatm Kctd14 Igfbp5 Col18a1 Pcsk6 Slc18a2 Fndc5 Wt1 Tmem184a Dipk2a
Inhba Nppc Mro Nap1l5 Hsd17b1 Slc26a7 Grem1 1110032F04Rik Cyp19a1 Tom1l1
Ghr Pik3ip1 Cald1 Itih5 Cfh Asb4 Ccn2 Ntn4 Cdc42ep3 Fhl1
Tinagl1 Adamts1 Mrap Prss35 Mt2 Parm1 Loxl2 S100a6 Cemip Cdkn1a
Neat1 Col3a1 Igfbp7 Sfrp4 Cyp11a1 Plin4 Onecut2 Col1a2 Col1a1 Gm42669
Ptgfr Efhd1 S100a6 Tnc Lipg Sfrp4 Lgmn Star Akr1c18 Sgk1
Preantral-Cumulus
Antral-Mural
Atretic
Luteinizing Mural
Active CL
Regressing CL

Supplemental Figure S5. Widespread gene expression changes induced by modest derepression of *Zeb1* in older female mice, related to Figure 3. Shown are analyses of RNA-seq results from WT and *Zeb1*^{200} mutant tissues from 28–36-week-old mice staged for proestrus at the conclusion of the breeding trial (*n* = 3–4 per genotype). Otherwise, this panel is as in Figure S3A–H.

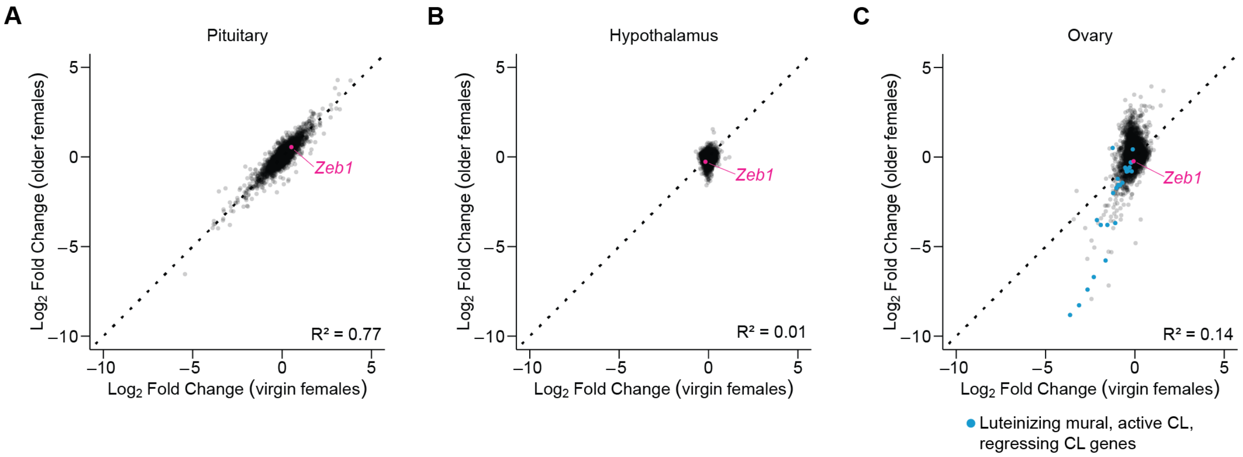

Supplemental Figure S6. Correspondence between RNA-seq analyses of two independent cohorts of mice, related to Figure 3. (A–C) Correlation of RNA fold changes observed between *Zeb1*^{200M} and WT pituitary (A), hypothalamus (B), and ovary (C). Plotted are mRNA fold changes, as determined by DESeq2. The correlation coefficient (Pearson R^2) is indicated. In panel (C), Granulosa marker genes from luteinizing mural, active corpus lutea (CL), and regressing CL are highlighted in blue.

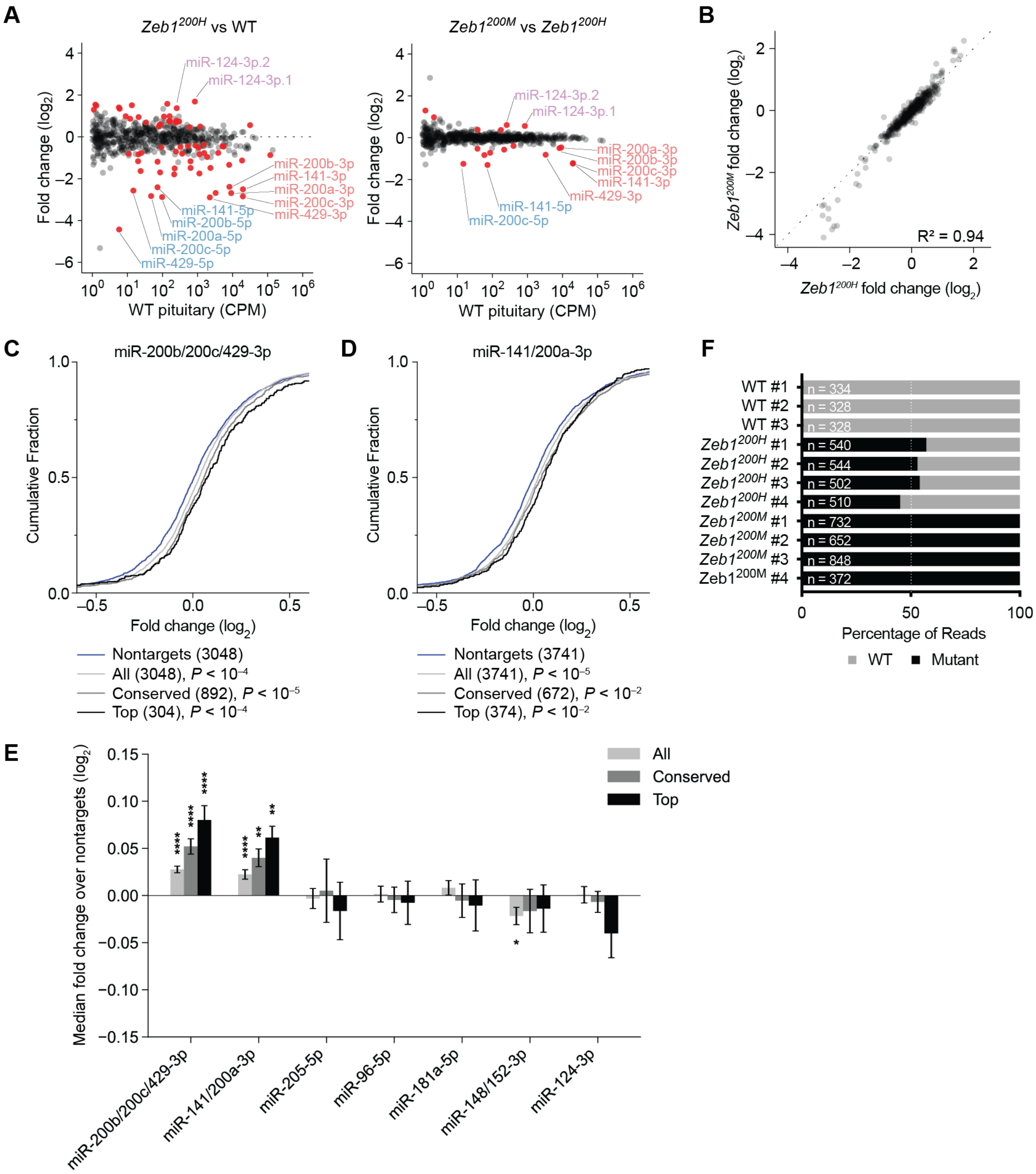

Supplemental Figure S7. Increased ZEB1 causes decreased expression of miR-200a/b and increased expression of miR-200a/b targets in virgin female mice, related to Figure 4. Shown are analysis of RNA-seq and small-RNA-seq, indicating miRNA expression and target repression in the pituitary of 8–12-week-old mice (*n* = 3–4 per genotype). (A) The influence of *Zeb1*^{200} alleles on mature miRNA levels in the

pituitary. Plotted are fold changes in mean miRNA levels for *Zeb1*^{200H} pituitary relative to wild-type pituitary (left) and *Zeb1*^{200M} pituitary relative to *Zeb1*^{200H} pituitary (right), as determined by small-RNA-seq and plotted as a function of expression in wild-type pituitary. Otherwise, as in Figure 4B. (B) Correlation of miRNA fold changes between *Zeb1*^{200} alleles. Otherwise, as in Figure 3D. (C–D) The influence of *Zeb1*^{200H} on expression of miR-200b/200c/429-3p and miR-141/200a-3p predicted targets. Otherwise, as in Figure 4D–E. (E) The influence of *Zeb1*^{200H} on expression of predicted targets of differentially expressed miRNAs. Otherwise, as in Figure 4F. (F) Allele-specific expression of *Zeb1* in wild-type and mutant pituitary. Plotted are the percentage of reads matching the wild-type or mutant miR-200a/b binding sites for wild-type, *Zeb1*^{200H}, and *Zeb1*^{200M} pituitary. Each bar represents a different animal, and the total number of reads are indicated in white.

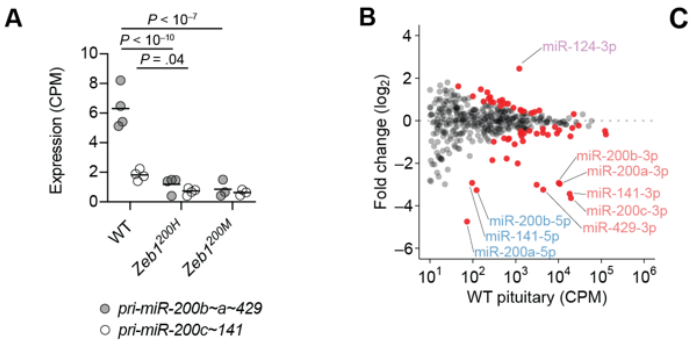

C

miRNA	WT pituitary (CPM)	*Zeb1*^{200H} fold change ($\log_2$)	*Zeb1*^{200M} fold change ($\log_2$)	Adjusted *p*-value ($-\log_{10}$)
miR-200c-3p	20000	−2.3	−3.6	244.1
miR-141-3p	18000	−2.4	−3.4	171.9
miR-200a-3p	11000	−2.8	−3.0	156.8
miR-200b-3p	10000	−2.6	−2.9	155.2
miR-429-3p	4400	−2.9	−3.2	104.3
miR-124-3p	1200	1.6	2.4	55.3
miR-205-5p	3100	−3.2	−3.0	53.6
miR-96-5p	1100	−1.6	−2.0	18.2
miR-335-5p	19000	−0.5	−0.7	9.5
miR-375-3p	130000	−0.6	−0.6	9.0
miR-182-5p	620	−1.9	−1.8	8.3
miR-152-3p	9200	−0.4	−0.6	6.3
miR-484	1500	−1.0	−1.0	6.3
miR-541-5p	4300	−0.2	−0.6	5.5
miR-544-3p	220	0.9	1.3	5.2
miR-181a-5p	5600	−0.6	−0.7	5.1
miR-140-5p	490	0.8	1.0	5.0
miR-540-5p	950	−0.7	−1.1	5.0
miR-34c-5p	150	1.3	1.5	4.6
miR-183-5p	290	−2.2	−1.9	4.2
miR-384-3p	660	0.4	0.8	3.9
miR-320-3p	3100	−0.2	−0.6	3.9
miR-330-5p	260	1.0	1.1	3.9
miR-7a-5p	120000	−0.2	−0.5	3.8
miR-30d-5p	10000	−0.3	−0.4	3.3
miR-200b-5p	120	−2.6	−3.3	3.2
miR-672-5p	1400	−0.3	−0.7	3.0
miR-326-3p	240	0.5	1.0	3.0
miR-335-3p	1600	−0.7	−0.6	3.0
miR-487b-3p	550	0.5	0.8	2.9
miR-384-5p	890	0.5	0.6	2.8
miR-142-3p	420	0.3	0.9	2.8
miR-34a-5p	660	−0.6	−0.9	2.7
miR-337-3p	700	−0.5	−0.8	2.7
miR-151-5p	1300	−0.3	−0.6	2.5
miR-135a-5p	3600	0.1	0.5	2.4
miR-141-5p	96	−3.5	−2.9	2.3
miR-200a-5p	73	−4.5	−4.7	2.3
miR-374b-5p	2100	0.0	−0.5	2.1
miR-3099-3p	260	0.9	0.9	2.1
miR-191-5p	4300	−0.2	−0.4	2.1
miR-9-5p	300	0.5	0.9	2.1
miR-137-3p	2800	0.1	0.4	2.1
miR-33-5p	440	0.3	0.7	2.0

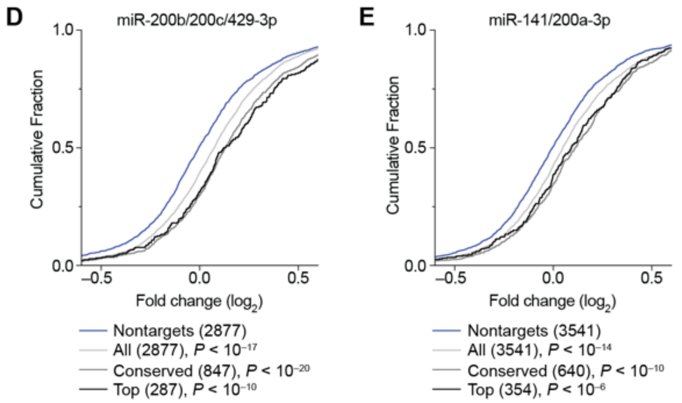

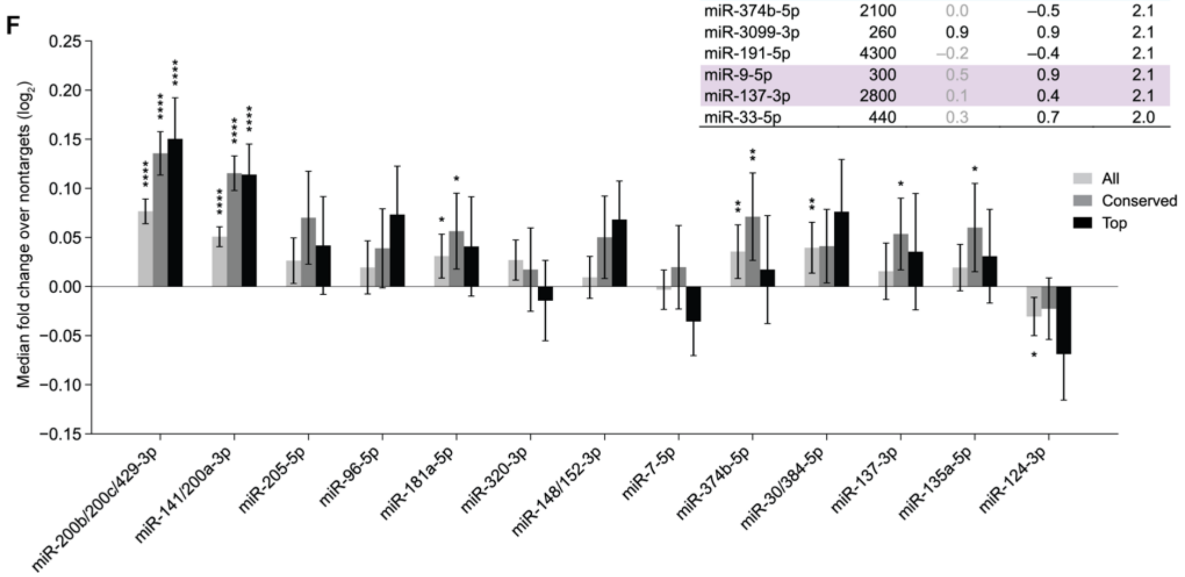

Supplemental Figure S8. Increased ZEB1 causes decreased expression of miR-200a/b and increased expression of miR-200a/b targets in older female mice, related to Figure 4. Shown are analyses of miRNA expression and target repression in the pituitary of 28–36-week-old mice staged for proestrus at the conclusion of the breeding trial (*n* = 3–4 per genotype). Otherwise, this panel is as in Fig. 4.

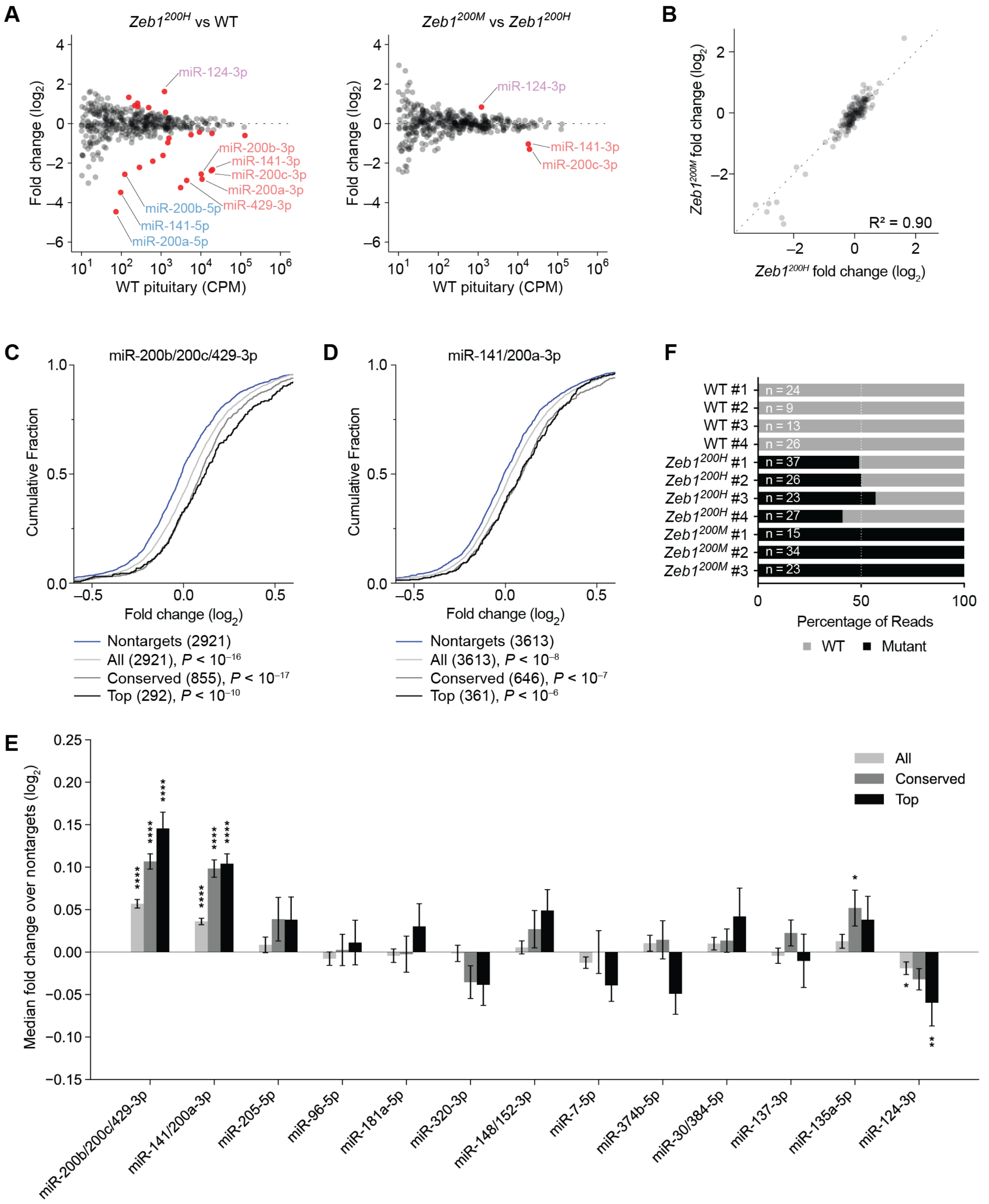

Supplemental Figure S9. Increased ZEB1 causes decreased expression of miR-200a/b and increased expression of miR-200a/b targets in older female mice, related to Figure 4. Shown are analyses of miRNA expression and target repression in the pituitary of 28–36-week-old mice staged for proestrus at the conclusion of the breeding trial (*n* = 3–4 per genotype). Otherwise, this panel is as in Fig. S7.

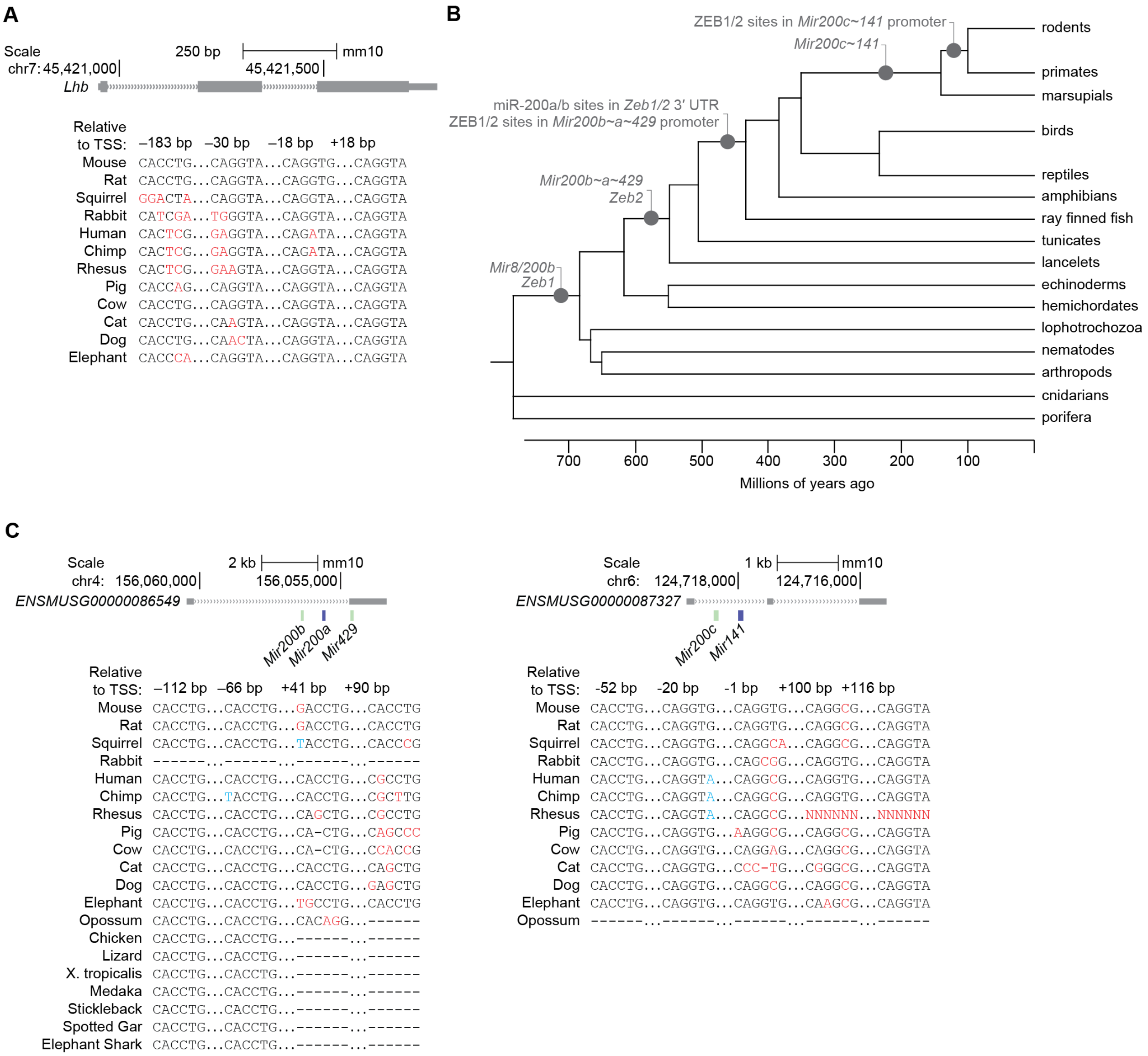

Supplemental Figure S10. Evolutionary conservation of the miR-200a/b–ZEB1/2 DNFL, related to Figure 5. (A) Organization of the murine *Lhb* locus. The *Lhb* gene model (gray boxes, exons; > > >, introns) is depicted. Diagramed below are four putative ZEB1/2 binding sites, their position relative to the mouse transcription start site (TSS), and Multiz alignments for each site in 12 placental mammal species (red denotes a nucleotide change that disrupts the CAGGTR consensus sequence). Not pictured are two additional human- or primate-specific CAGGTR motifs located at –604 bp and +152 bp relative to the human TSS. (B) Phylogeny of Metazoa, indicating the most parsimonious emergence of genes and regulatory sequences in the miR-200a/b–ZEB1/2 DNFL. (C) Organization of the murine *Mir200b~a~429* (left) and *Mir200c~141* (right) loci. The transcript model of the presumptive murine host gene for each miRNA cluster (gray boxes, exons; > > >, introns) is depicted with the position of the miRNA hairpins shown below (green and blue boxes). Diagramed below that are four or five putative ZEB1/2 binding sites, their position relative to the mouse TSS, and Multiz

alignments for each site in 13 mammals and, for *Mir200b~a~429*, seven more deeply branching vertebrates (red denotes a nucleotide change that disrupts the CAGGTR consensus sequence, blue denotes a nucleotide change that does not disrupt the CAGGTR consensus).
